# Hyperplastic ovarian stromal cells express genes associated to tumor progression: a case study

**DOI:** 10.1186/s12917-024-04275-6

**Published:** 2024-09-28

**Authors:** Arpna Sharma, Frank Becker, Xuelian Tao, Vijay Simha Baddela, Dirk Koczan, Carolin Ludwig, Jens Vanselow

**Affiliations:** 1 Forschungsinstitut für Nutztierbiologie (FBN), Wilhelm-Stahl-Allee 2, 18196 Dummerstorf, Germany; 2 Institut für Immunologie, Universität Rostock, 18055 Rostock, Germany; 3 Department of Animal Sciences, University of Illinois Urbana-Champaign, Urbana, USA

**Keywords:** Ovary, Stroma, Hyperplastic

## Abstract

The current study presents the analysis of stromal cells obtained from an hyperplastic left-ovary of a Holstein cow. Cultured hyperplastic stromal cells displayed a fibroblast-like morphology and ceased proliferation after the 8th passage. The non-cancerous nature of stromal cells was confirmed by in vitro cell proliferation and migration assays. Negligible amounts of E2 were detected in the spent media of cultured stromal cells, which suggests that stromal cells were non-estradiol synthesizing cells. As revealed in immunofluorescence and gene expression analysis, the hyperplastic stromal cells explicitly expressed vimentin in their cytoskeleton. Upon hematoxylin staining, a highly dense population of stromal cells was observed in the stromal tissue of the hyperplastic ovary. To explore genome-wide alterations, mRNA microarray analysis was performed using Affymetrix Bovine Gene 1.0ST Arrays compared to normal ovarian derived stromal cells. The microarray identified 1396 differentially expressed genes, of which 733 were up- and 663 down-regulated in hyperplastic stromal cells. Importantly, asporin (*ASPN)* and vascular cell adhesion molecule 1 (*VCAM1)* were among the highly up-regulated genes. Higher expression of *ASPN* was also confirmed by immunohistochemistry and RT-qPCR analysis. Ingenuity pathway analysis (IPA) identified about 98 significantly enriched (-log (*p value* ≥ 1.3) canonical pathways, importantly of which the “Sirutin Signaling Pathway” and “Mitochondrial Dysfunction” were highly activated while “Oxidative phosphorylation” was inhibited. Additionally, higher proportion of hyperplastic stromal cells in the S-phase of cell cycle, could be attributed to higher expression levels of cell proliferation genes such as *CCND2* and *CDK6*.

**Supplementary Information:**

The online version contains supplementary material available at 10.1186/s12917-024-04275-6.

## Introduction

The bovine ovary consists primarily of two types of tissues such as (1) specialized parenchymal tissue enclosing numerous growing follicles containing oocytes and (2) supportive connective tissue, commonly referred as ovarian stroma. The ovarian stroma consists of a heterogeneous population of cells along with incompletely characterized stromal cells such as fibroblast-like spindle cells and interstitial cells [1]. In the ovarian stroma, fibroblast-like stromal cells are arranged in a characteristic whorled texture [2]. There has been a great interest in identifying and understanding the functions of these incompletely characterized stromal cells that largely produce collagen, a vital part of the extracellular matrix responsible for overall cell support, adhesion, and motility [3–6]. Non-neoplastic proliferation of stromal cells in the ovary can result into stromal hyperplasia [7]. Ovarian stromal hyperplasia has been reported in post-menopausal women with densely populated cells in the ovarian cortical stroma [8]. Interestingly, reports on mutant mice models displaying hyperplasia in ovary, mammary gland and uterus were found to be more susceptible to tumor development [9]. Additionally, recent findings in humans suggest that ovarian stroma is a major source of cancer-associated fibroblast cells (CAFs) [10]. Though, cases of ovarian stroma derived tumors in cattle are very rare, previous study revealed a case of an ovarian vascular hamartoma, a non-neoplastic tumor-like abnormality of vascular origin has been detected [11]. Another case study revealed a granulosa cell tumor (GCT) with estrogen receptor beta (Erβ) expression [12] and increased plasma concentration of anti-Müllerian hormone (AMH) [13]. Similarly, in the case of a bovine ovarian sex cord–stromal tumor, the cut section of the tumor tissue revealed a highly tubular structure and high plasma levels of AMH, inhibin and 17β-Estradiol (E2) [14]. However, none of these case studies have examined the in vitro morphology or altered physiological state of cells isolated from the reported tumor tissue. Therefore, in our study, we performed an in vitro analysis of stromal cells isolated from the hyperplastic left-ovary and examined if such enlarged ovarian tissue is associated to tumor progression.

### Case description

**Fig. 1:**
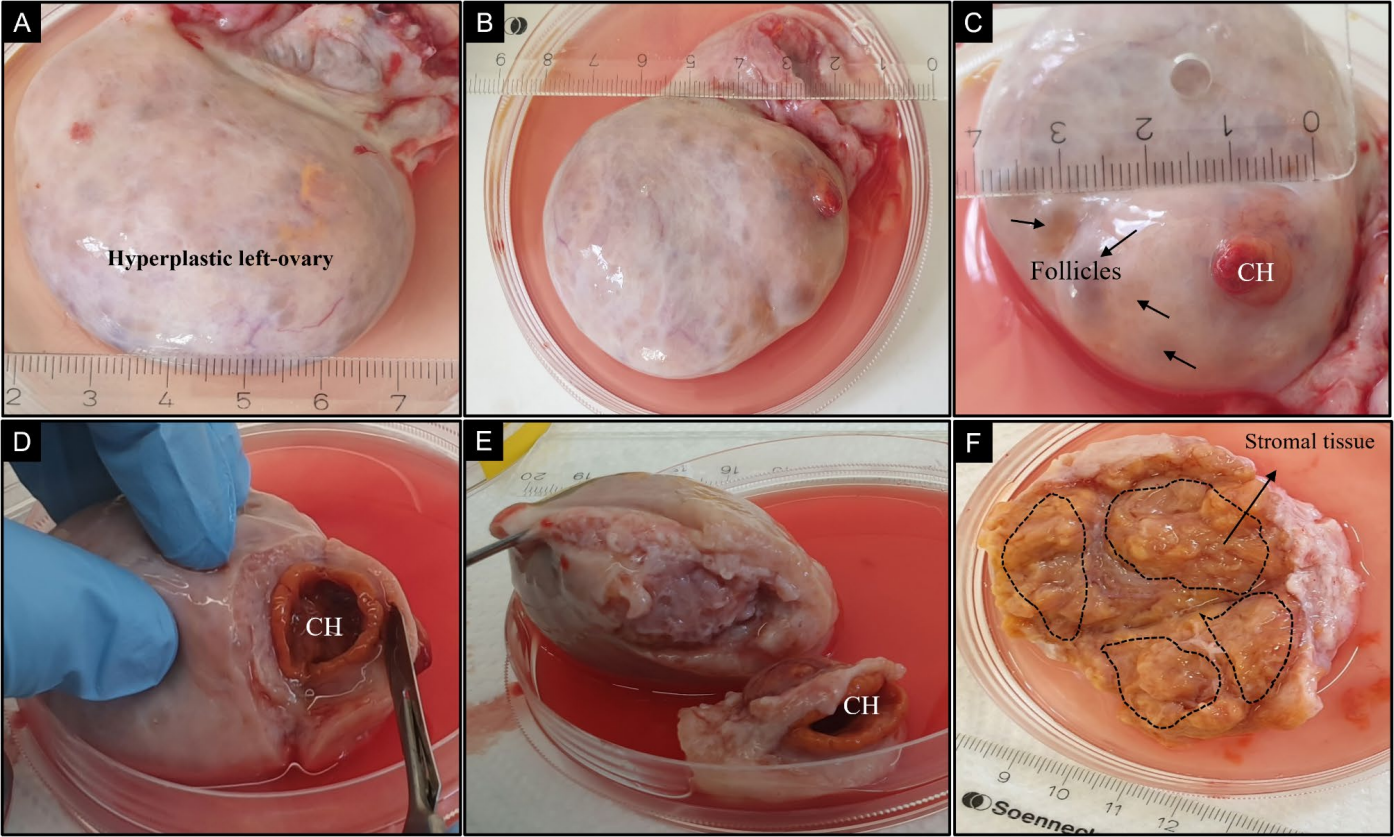
**(A-B)** Excised enlarged left-ovary of approx. 7 cm length and 4.5 cm in diameter. **(C)** The excised abnormally enlarged left-ovary showed a clear corpus hemorrhagicum (CH) and small follicles. **(D-E)** The CH was cut-off aside and remaining enlarged ovary was cut into halves revealing the **(F)** uniformly loose packed yellowish stromal tissue. The demarcated dashed region shows the region from which stromal tissue was picked for cell preparation

Following the unsuccessful artificial insemination in 2021, a Holstein-Friesian cow (2nd lactation, 9645 kg milk yield in 305 days, 767 kg body weight) was diagnosed with an enlarged left-ovary with a length of approximately 7 cm and 4.5 cm diameter (Fig. 1A). No further changes in structure, shape and size were observed during the weekly veterinarian examination. The ovary was interspersed with hyper-echogenic and anechogenic trabecular structures. Administration of gonadotropin-releasing hormone (GnRH) induced ovulations on both the ipsi and contralateral ovaries. Likewise, the developed corpus luteum (CL) responded to prostaglandin F2α (PGF2α) treatment by undergoing luteolysis. During this time, no new artificial insemination was performed. In addition to the circumferential increase in size, the left-side ovary also showed a developing CL and some small follicles (Fig. 1B-C). Finally, as a therapeutic measure and for diagnostic purposes, an ovariectomy of the left-ovary was carried out. Due to the abnormally enlarged size of the left-ovary the preliminary assumption was of tumor progression. For a detailed in vitro analysis the left-ovary was sliced and cells from large ovarian stromal tissue were isolated.

## Materials and methods

### Isolation and culture of hyperplastic ovarian stromal cells (HOSC)

The Holstein cow was owned, handled and treated in accordance to general guidelines and regulations approved by the Forschungsinstitut für Nutztierbiologie (FBN) authority. For the diagnostic purposes of hyperplastic left-ovary detected in the Holstein cow, an ovariectomy of left-ovary was carried out. The hyperplastic left-ovary was washed, rinsed in phosphate buffered saline (PBS) and the corpus hemorrhagicum was removed (Fig. 1D-E). The ovary was then sliced into halves, revealing a uniformly distributed loosely held stromal tissue (Fig. 1F). Using a sterile scalpel, stromal tissue pieces of 1–3 mm were excised from the inner medullary region and digested enzymatically to isolate the cells. Briefly, small sliced stromal tissues were transferred in a 20 ml digestion solution containing HBSS-Hepes + Antibiotic and 200 µl collagenase (0.1%) and digested for 25 minutes (min.) on a thermal shaker at 37 °C. This step was repeated with undigested tissue chunks at least 4 times to completely disintegrate the sliced stromal tissue sections into single cells. Finally, the digestive solution containing single cells was passed through a cell sieve of 100 μm to remove all the undigested residual tissue. The obtained single cell suspension was centrifuged at 400 g for 5 min. and later the cell pellet was re-suspended in DMEM/HAM’s F12 containing 10% fetal bovine serum (FBS) (Bio&Sell, Germany) culture media and was subjected to in vitro culture in a humidified 37 °C, 5% CO_2_ incubator. The cultured cells were examined for the next 2–3 days and all floating unattached cells were removed by washing with 1x PBS and the media was replaced every third day. The attached cell monolayer displayed a pure population of fibroblast-like morphology in vitro. In culture, hyperplastic ovarian stromal cells (HOSC) were passaged until the cell proliferation in vitro reached a cessation point. Parallely, in order to compare the in vitro morphology and functions of isolated HOSC, ovaries (n=4) from local abattoir were used for isolating normal ovarian stromal cells (NOSC) and performing histological analysis as well. NOSC isolated from normal ovaries served as a control to HOSC. Initially, NOSC were isolated with a similar procedure as HOSC, however isolation of NOSC resulted in a mixed population of stromal fibroblast cells and epithelial cells. To overcome this, small sliced pieces of normal stromal tissues were digested with collagenase followed by directly placing the tissue explant in a culture plate along with culture media and left to grow. After 3–4 days, culture plates were examined for cells growing out from the explant. Subsequently, cells reaching 70–80% confluency were released from the culture dish by accutase treatment and were then plated onto six-well culture dishes. At subsequent passages, a substantial population of NOSC was obtained and if any patches of epithelial cells occurred, they were discarded by scraping with a sterile scraper with subsequent culture medium change. After cell isolation, the cells (HOSC/NOSC) were cryopreserved in freezing medium (10% DMSO + 90% FBS) and stored in liquid nitrogen (LN2).

### Radioimmunoassay (RIA)

To examine if the HOSC were steroidogenic cells, the concentration of E2 and progesterone (P4) in spent culture media of both NOSC and HOSC were determined by competitive 3 H–radioimmunoassay with rabbit-raised antibodies purified by affinity chromatography. The E2 tracer (2, 4, 6, 7–3 H estradiol-17β) (GE Healthcare (Freiburg, Germany) and P4 tracer [1, 2, 6, 7 –3 H (N)] progesterone (PerkinElmer, Boston, USA) were used for E2 and P4 estimation, respectively. Assay standards were prepared in RIA buffer after dissolving the tracers in 100% ethanol. Spent media for P4 estimation was diluted in a ratio of 1:20 in RIA buffer and used undiluted for E2 estimation. The radioactivity levels were measured in a liquid scintillation counter (LSC) with an integrated RIA-calculation programme (TriCarb 2900 TR; PerkinElmer, Germany).

### Cell viability assay

NOSC and HOSC cells in their logarithmic growth phase were harvested and approximately 10,000 cells were seeded in 100 µl of DMEM/HAM’s F12 (10% FBS) medium in each well of a 96well plate in triplicates. 20 µl of CellTtiter 96^®^ AQueous one solution Cell Proliferation Assay (MTS) reagent (Promega, G358A) was added to each well of cultured cells and incubated for 3–4 h. Finally, absorbance was measured, at 490 nm (FLUOstar Omega) at regular intervals of 24, 48, 72, 96, 120 and 144 h.

### Cell cycle analysis

The proportion of proliferating cells was determined by the amount of DNA fluorescence detected by flow cytometry. Briefly, NOSC and HOSC at passage 3 were washed with 1xPBS and subjected to trypsinization by adding 800 µl accutase (Sigma-Aldrich, A6964) to each well of the 6-well culture plate. The detached cells were centrifuged (3 min., 500 g) and the remaining cell pellet was dissolved in 300 µl of 1x PBS. Next, the cell suspension was slowly added dropwise into 70% ice-cold ethanol and stored at -20 °C. Later, cells were pelleted (300xg, 10 min., 4 °C), re-suspended in 1 ml RNase solution (1 mg/ml), and incubated at 37 °C for 30 min. Next, propidium iodide (PI) reagent (500 µg/ml) was added to the cells and incubated in the dark at 37 °C for 30 min. Lastly, the fluorescence signal was quantified from single cells (10,000 counts) by a flow cytometer (Gallios, Beckman-Coulter, Krefeld, Germany) and proportions of cells at different cell cycle phases were analyzed using the MultiCycle Tool of FCS Express software.

### Cell migration assay

The cell migration assay was performed to analyze and compare the wound healing and migration pattern of NOSC and HOSC *in vitro.* Approximately 1.1 × 10^4^ cells were seeded in each well of the Culture-insert 2 Well in µ-Dish (35 mm) high (ibidi, catalog.no:81176). Once the cells reached 80% confluency, culture insert was removed, creating a cell free gap of 500 μm. Next, the cell monolayer was gently washed with 1x PBS to remove any detached cells and then fresh DMEM/HAM’s F12 (10% FBS) medium was added. Images were captured under phase contrast microscope at 10x magnification at different time intervals on the same cell free gap of 500 μm each time at 0, 24, 48 and 168 h.

### Hematoxylin and eosin (H&E) staining

Stromal tissue sections from both a normal and the hyperplastic ovary were sliced into small pieces for fixation in bouin’s reagent (10 ml of 37% formalin, 50 ml of glacial acetic acid and 150 ml of picric acid) for two days. Tissue slices were then dehydrated serially in a gradient of ethanol and subjected to paraffin embedding using an MPS/W instrument (SLEE medical GmbH, Germany). 3–5 μm tissue sections were prepared using a microtome and mounted onto glass slides and stored until use. For H&E tissue staining tissue slides were deparaffinized by heating for 45 min. at 60 °C. Followed by 2 × 5 min. immersion in roti clear solution, 2 × 3 min. in 99% isopropanol and 1 × 3 min in 70% isopropanol, 50% isopropanol and distilled water respectively. Next, tissue slides were stained with hematoxylin (Roth) for 3 min. followed by 2 min. washing in water. Followed by 1 min of staining in eosin-G (0.5%) (Roth X883.2) and 1 min of washing with water. Finally, stained tissue slides were mounted with roti-mount (Roth 2848.2) dried and scanned under a bright field Axio imager A1 microscope (Carl Zeiss Inc, Germany) for imaging the internal tissue structures.

### Cell immunofluorescence

To characterize the NOSC and HOSC, intracellular localization of major intermediate filament proteins such as vimentin and cytokeratin-18 were evaluated by immunofluorescence. Cells were cultured in µ-Slide 8 Well high chamber slides (ibidi, cat.no:80806) for 2–3 days and at 70–80% confluency cells were fixed in 4% paraformaldehyde (PFA) for 21 min. at 4 °C, next washed in 1x PBS, permeabilized with 0.1% Triton X-100 for 10 min. at room temperature (RT) and blocked with 5% bovine serum albumin (BSA) for 30 min. at RT. Further, cells were incubated with primary antibodies against vimentin (Invitrogen, catalog no. MA5-11883, 1:100) and cytokeratin-18 (Novus biologicals, catalog no. NBP2-44951, 1:100) at 4 °C for overnight. The next day, cells were rinsed four times in wash buffer and were incubated with goat anti-Mouse IgG (H + L) cross-adsorbed secondary antibody, Alexa Fluor 647 (Invitrogen, catalog no. A21235, 1:200) in the dark at RT for 1 h. Further, cells were washed 4 × 5 min. with wash buffer to omit excess antibodies. Next, cells were incubated with SYBR green (1:500) in the dark at RT for 20 min. followed by 4x rinsing in wash buffer and further incubation in 2% PFA at RT for 20 min. Additionally, bovine mammary alveolar cells-large T antigen (MAC-T) cell line was used as positive control cells to validate the cytokeratin-18 staining (1:50/1:100). Lastly, cell fluorescence was visualized at 20x oil objective lens and images were captured by confocal laser scanning microscope LSM 800 assembled with ZEN software (Carl Zeiss Inc, Germany).

### Microarray analysis and validation by real time-quantitative PCR (RT-qPCR)

Total RNA was extracted from NOSC and HOSC at passage 3 and global gene expression profiles were analyzed using GeneChip™ Bovine Gene 1.0ST Array (Affymetrix^®^, Inc., Santa Clara, CA, United States). RNA integrity was measured in all samples by bio-analyzer with RIN value of 10 displaying intact RNA (Supplementary image 1). GeneChip 3´amplification and one-cycle target labeling reagents were used for amplification and labeling of RNA samples. RNA samples and probes were hybridized overnight in a hybridization oven followed by attainment of the gene expression signals using an Affymetrix Gene Chip Scanner 3000. Normalization and background reduction of gene expression were executed by the robust multichip average method. The gene expression data obtained were further analyzed using the TAC 4.0 software (Transcriptome Analysis console 4.0, Affymetrix). Differentially expressed (DE) genes were recognized using the cut-off criteria, fold change (FC) ≤**-**2 or ≥ 2, ANOVA, *p* < 0.05, and false discovery rate (FDR) *p* < 0.05. Simultaneously, NOSC and HOSC at passage 3 were subjected to RNA isolation using the innuPREP RNA Mini Kit (Analytik Jena, Germany) according to the manufacturer’s protocol and RNA concentration was quantified with NanoDrop1000 Spectrophotometer (Thermo Scientific, Bonn, Germany). Later, cDNA was synthesized using the SensiFAST cDNA synthesis Kit (Bioline, Luckenwalde, Germany) from 200 ng of RNA. Further, gene expression was analyzed by RT-qPCR using SensiFAST SYBR No-ROX (Bioline) with gene-specific primers (Supplementary sheet 1) in a light cycler 96 instrument (Roche, Mannheim, Germany). The abundance of gene transcripts were normalized using TATA-binding protein (*TBP*) as house-keeping gene.

### Immunohistochemistry (IHC)

IHC was performed to localize the presence of asporin protein in hyperplastic stromal tissue sections. Small stromal tissue sections from both a normal and the tumorous ovary were prepared as already mentioned earlier. For immunolocalization, tissue sections were deparaffinized and blocked with 2% BSA for 1 h. at RT and incubated with either anti-asporin antibody (Thermofisher, catalog no. PA5-18352; 1:100) in 1% BSA or 1% BSA (without antibody) as control overnight. Next day, slides were washed four times with wash buffer and incubated with rabbit anti-Goat IgG (H + L) secondary antibody, HRP, 1:2000 (Thermofisher, catalog no. 31402) for 1 h at RT. Subsequently, slides were then washed four times in wash buffer and subsequently treated with DAB (Roti-DAB-Kit, Roth 9202.1). All slides were counterstained with Mayers hemalum solution for 10 s followed by 2 min washing in water. Finally, slides were secured by adding roti-mount-aqua solution for color protection and images were obtained using a bright field with Axion imager A1 microscope (Carl Zeiss Inc, Germany).

### Bioinformatics and statistical analysis

Ingenuity Pathway Analysis software (IPA; Qiagen Bioinformatics software solutions) was used to identify affected canonical pathways, upstream regulators and diseases and functions. Hub genes were recognized by constructing a protein-protein interaction (PPI) network using web based network analysis tool available at www.networkAnalyst.ca. Gene ontology (GO) terms was performed using WebGestalt (WEB-based GEne SeT AnaLysis Toolkit) a web based gene set analysis tool. The RT-qPCR mRNA abundance, hormone concentration, flow cytometry and cell viability values (three independent experiments) were subjected to unpaired *t-test* and Mann-Whitney Rank Sum test where ever required in SigmaPlot 11.0 to test for any statistical significance, and graphical images were created using ggplot2 in R package. All statistically significant changes were recognized if *p* < 0.05.

## Results

### HOSC showed limited proliferation in vitro and were estrogen inactive

**Fig. 2:**
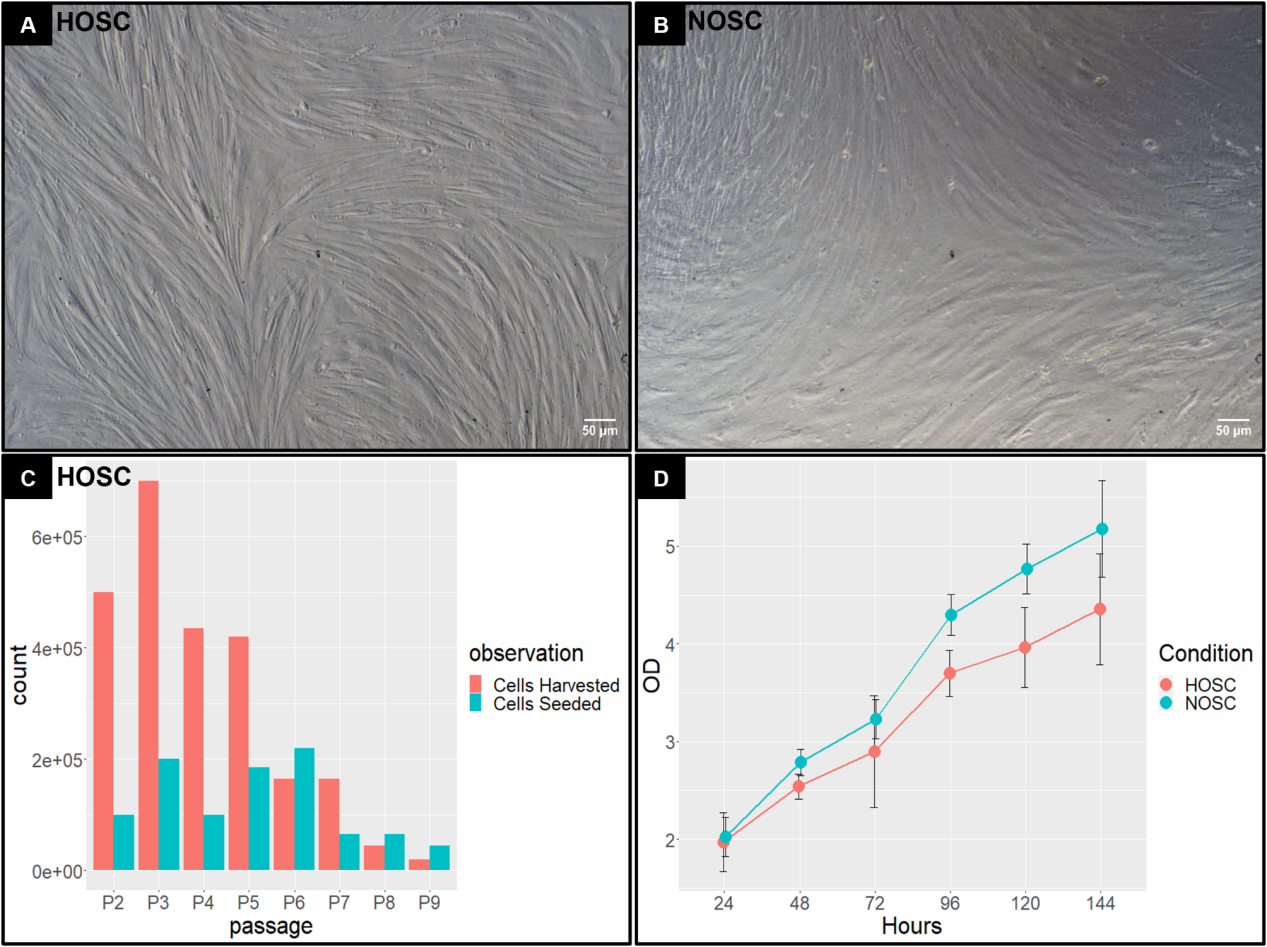
In vitro cultured **(A)** HOSC and **(B)** NOSC displaying spindle shaped, fibroblast-like morphology, scale bar 50 μm **(C)** HOSC at different passages (2–9); cells seeded = no. of cells initially cultured; cells harvested = no. of cells collected at the end of culture period at each passage **(D)** Cell growth curve of NOSC and HOSC representing different time points (24, 48, 72, 96, 120 and 144 h.) at x-axis and measured absorbance (490 nm) at y-axis. Statistical significant changes were acknowledged with unpaired *t-test*, if *p* < 0.05. Data represented as means ± SEM, n=3

Cultured HOSC displayed spindle shaped, fibroblast-like morphology (Fig. 2A). A similar morphology was also shown by NOSC in vitro (Fig. 2B). The subsequent sub-cultured HOSC showed a steep decline in proliferation as cells reached the 8th passage (Fig. 2C). This clearly indicated that HOSC possessed a definite life span in vitro and were not undergoing any malignant transformation. The cell viability assay showed no significant difference between the absorbance values for NOSC *versus* HOSC at all tested time points (Fig. 2D). This indicates that HOSC possessed similar viability kinetics and were equally viable as compared to NOSC. In addition, to test the possibility of a GCT or if HOSC were synthesizing E2, the spent media of cultured HOSC was subjected to RIA. Results revealed negligible amounts of E2 in spent media of both NOSC (0.63 ± 0.024 ng/ml) and HOSC (0.60 ± 0.053 ng/ml) while low and approximately similar amounts of P4 were observed in spent media of both NOSC (46.41 ± 2.22 ng/ml) and HOSC (48.71 ± 2.3 ng/ml). Together, these results indicated that HOSC displayed a strikingly different morphology in vitro and were neither E2 nor P4 synthesizing cells unlike granulosa or luteal cells that secrete huge amounts of E2 and P4 in vitro, respectively (supplementary image 2).

### HOSC showed contact inhibition in vitro

**Fig. 3:**
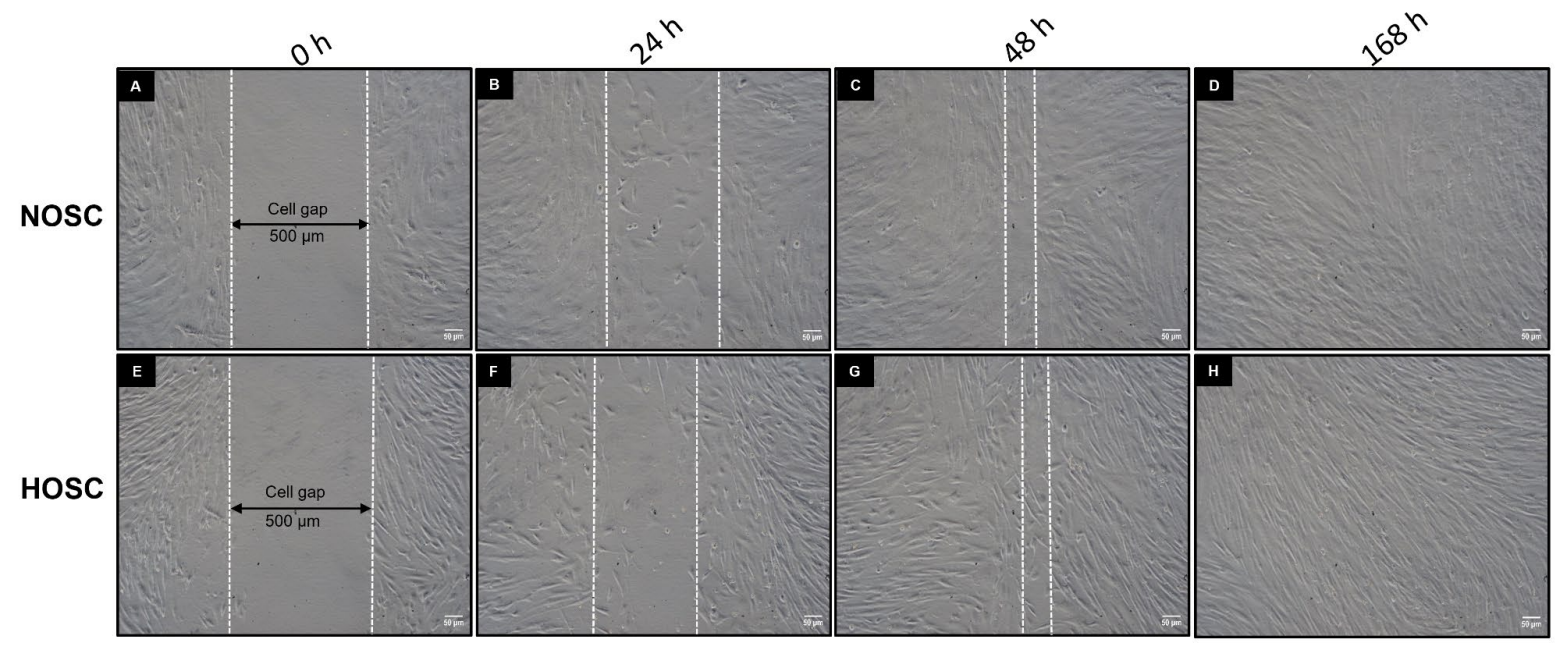
Cell migration assay: **(A-D)** NOSC and **(E-H)** HOSC cultured in insert wells with a cell free gap of 500 μm. Migration of cells across the same gap area was captured under phase contrast microscope (10x) at different time points (0, 24, 48 and 168 h.). The white dotted lines defines the migrating edges of cells, scale bar 50 μm

Since, we preliminary suspected the excised ovary an ovarian tumor, so we tested the hyperplastic stromal cells for tumor cell properties such as migration and wound healing. Metastatic tumor cells have very low wound healing capability [15] and are not inhibited by cell contact, so they migrate over one another and grow in a disordered, multilayered pattern [16]. The migration behaviors of both NOSC and HOSC were therefore analyzed at different time points (0, 24, 48 and 168 h) across a 500 μm cell free gap created in an 80% confluent cell layer. Microscopic images of NOSC (Fig. 3A and D) and HOSC (Fig. 3E and H) during migration were captured at a 500 μm cell free gap at every time point. Microscopic observations showed that both NOSC and HOSC migrated at almost similar rate to close the gap in the monolayer until they made contact with the neighboring cells. Further cell migration was inhibited and cells adhered to each other forming a normal orderly side-by-side array on the culture surface.

### HOSC positively expressed mesenchymal cytoskeletal markers

**Fig. 4:**
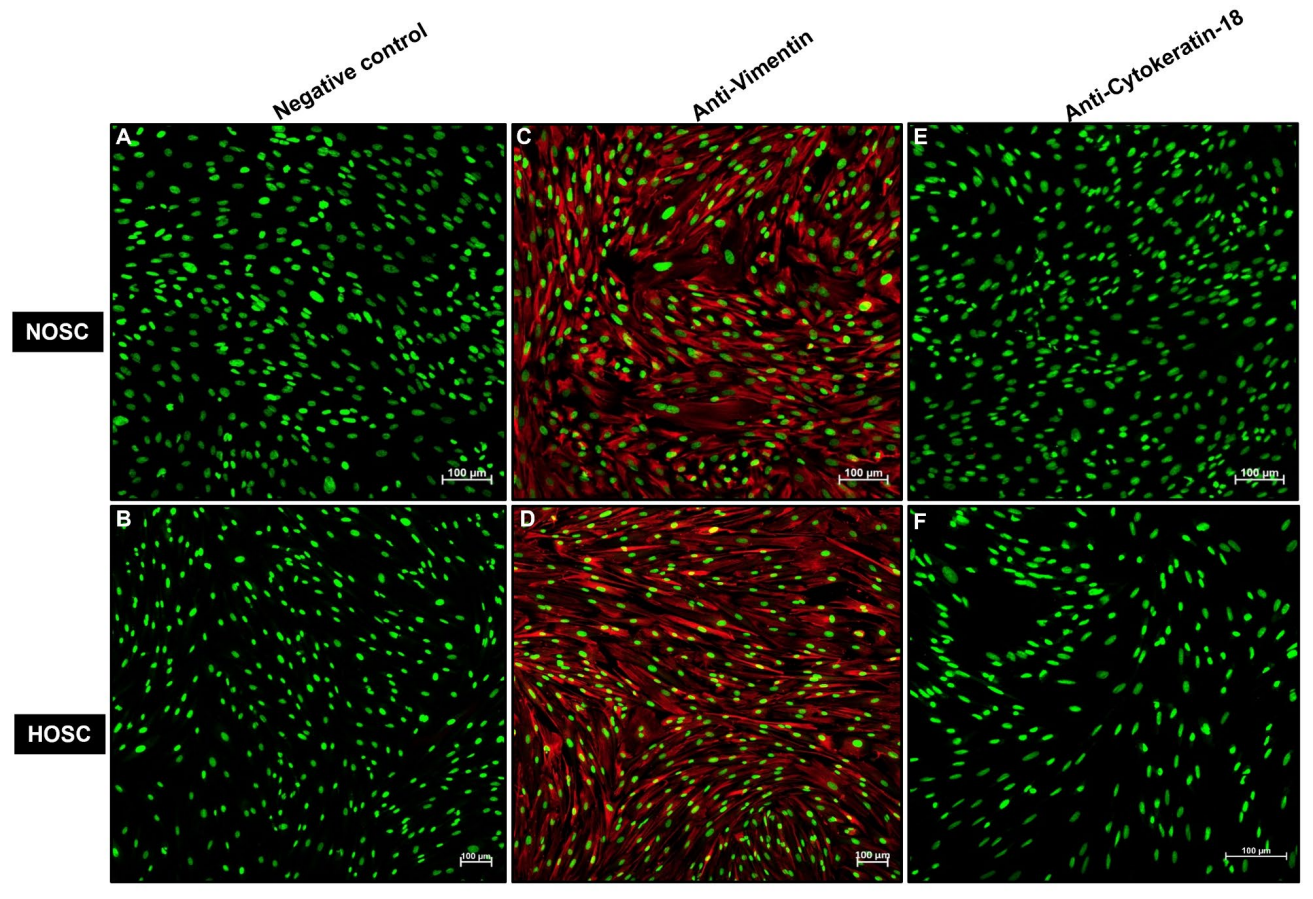
Representative cell immunofluorescence of ovarian stromal cells. Both NOSC and HOSC expressed vimentin, a mesenchymal cell cytoskeleton protein. **(A)** NOSC; negative control (without primary antibody), (**B)** HOSC; negative control (without primary antibody), (**C)** NOSC with vimentin expression, (**D)** HOSC with vimentin expression and (**E)** (**F)** showing absent cytokeratin-18 expression in NOSC and HOSC respectively. Fluorescent images were captured at 20x magnification under LSM fluorescent microscope, scale bar 100 μm

Cultured NOSC and HOSC were characterized for the expression of cytoskeleton intermediate filaments vimentin and cytokeratin-18 as mesenchymal and epithelial marker proteins, respectively, by immunofluorescence. The negative control for NOSC (Fig. 4A) and HOSC (Fig. 4B), without any included primary antibody showed only SYBR green fluorescence in cell nuclei. Further, both NOSC (Fig. 4C) and HOSC (Fig. 4D) explicitly expressed vimentin in their cytoskeleton. In addition, cytokeratin-18 expression was absent in both cell types NOSC (Fig. 4E), or HOSC (Fig. 4F). However, cytokeratin-18 was positively expressed in positive control bovine MAC-T cells (Supplementary image 3).

### Higher proportion of HOSC in S-phase of cell cycle defines over-amplified stromal cell population in ovarian stroma

**Fig. 5:**
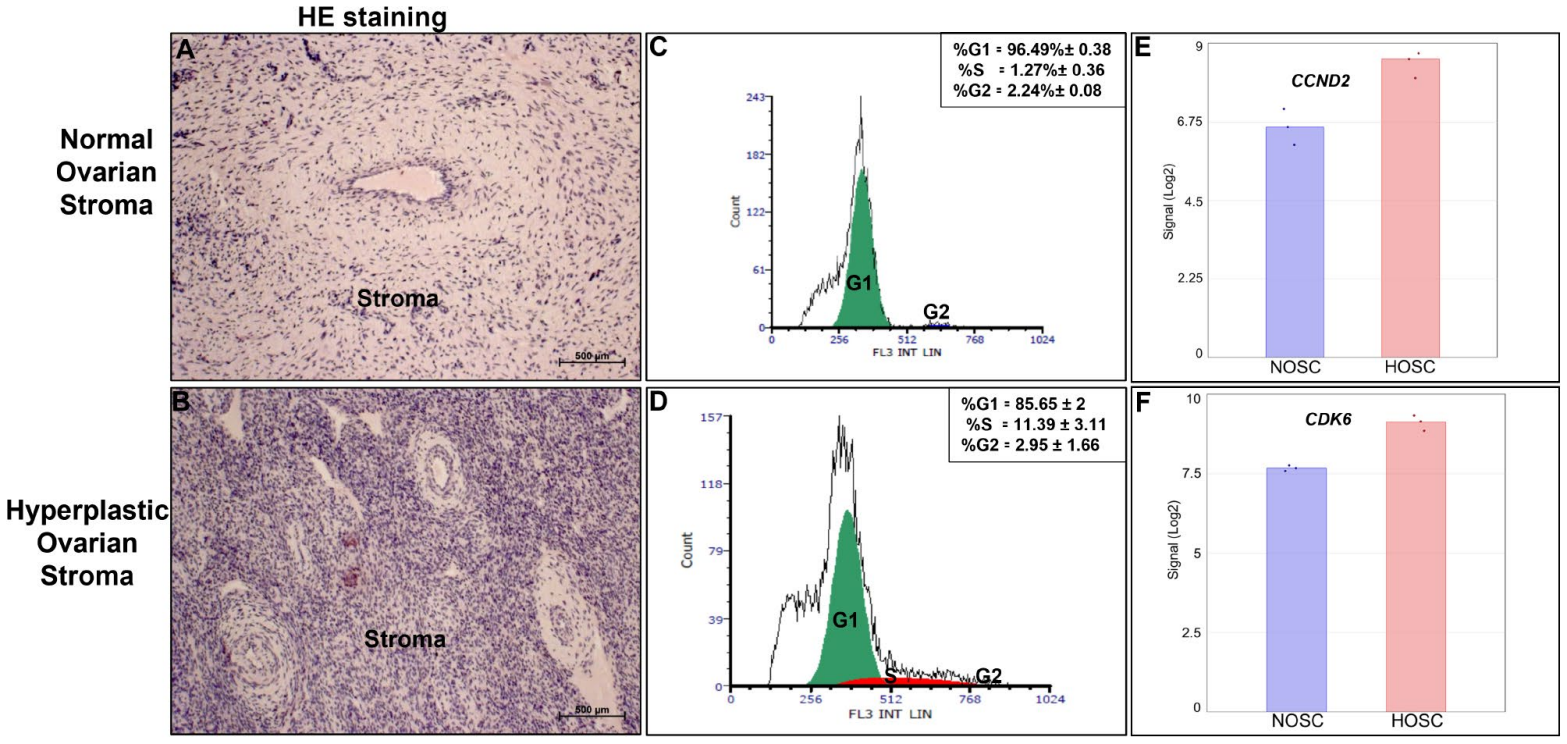
H&E tissue staining: **(A)** Normal ovary stroma **(B)** Hyperplastic ovarian stroma. Cell nuclei stained by hematoxylin bluish purple and eosin stained the cytoplasm and extracellular matrix in pink shade (scale bar 500 μm). Fluorescent DNA content estimation by flow cytometry: **(C)** Representative cell cycle distribution in NOSC and **(D)** HOSC. Numbers indicate average percentage (%) of cells in G1, S and G2-phase of cell cycle; green peak: G1-phase, red peak: S-phase of cell cycle and blue peak: G2-phase. Data represented as % means ± SEM, n=3. **(E)** Differential gene expression signal of *CCND2* (*q value*=0.0002) and **(F)***CDK6* (*q value*=0.0013) between NOSC and HOSC as revealed by microarray analysis. Statistical significant changes in DE genes were acknowledged with ANOVA, FDR *p* < 0.05

Normal and hyperplastic stromal tissue sections were treated with hematoxylin, which stained the cell nuclei purple-blue, and with eosin, which stained the extracellular matrix and cytoplasm with a light shade of pink. As evident from the results compared to normal stromal tissue (Fig. 5A), the hyperplastic stromal tissue clearly showed a highly dense population of stromal cells with purple-blue stained nuclei (Fig. 5B). This suggests that hyperplastic stroma carried an unusually amplified population of stromal cells with certainly altered but not uncontrolled proliferation behavior as observed in aggressive tumors. Further, flow cytometry was used to estimate the percentages of NOSC and HOSC cell populations in different cell cycle phases. The data showed, on average a lower percentage (85.65%±2) of HOSC in G1-phase as compared to NOSC (96.49%±0.38). However, compared to NOSC (1.27%±0.36) (Fig. 5C), a statistically (*p value*=0.032) higher proportion of HOSC were observed in S-phase (11.39%±3.11) (Fig. 5D) of the cell cycle. The proportion of NOSC (2.24%±0.08) and HOSC (2.95%±1.66) in G2-phase were indifferent. Additionally, the cell proliferation activation genes cyclin D2 (*CCND2) (q value =0.0067)* and cyclin dependent kinase 6 (*CDK6) (q value =0.0013)* were found to be significantly higher in HOSC as compared to NOSC as indicated in microarray data (Fig. 5E and F). Thus, higher proportions of HOSC in S-phase of the cell cycle and activation of cell proliferation genes in HOSC indicates towards a possible over-amplified population of stromal cells in the hyperplastic ovary.

### Microarray data analysis and RT-qPCR validation

**Fig. 6:**
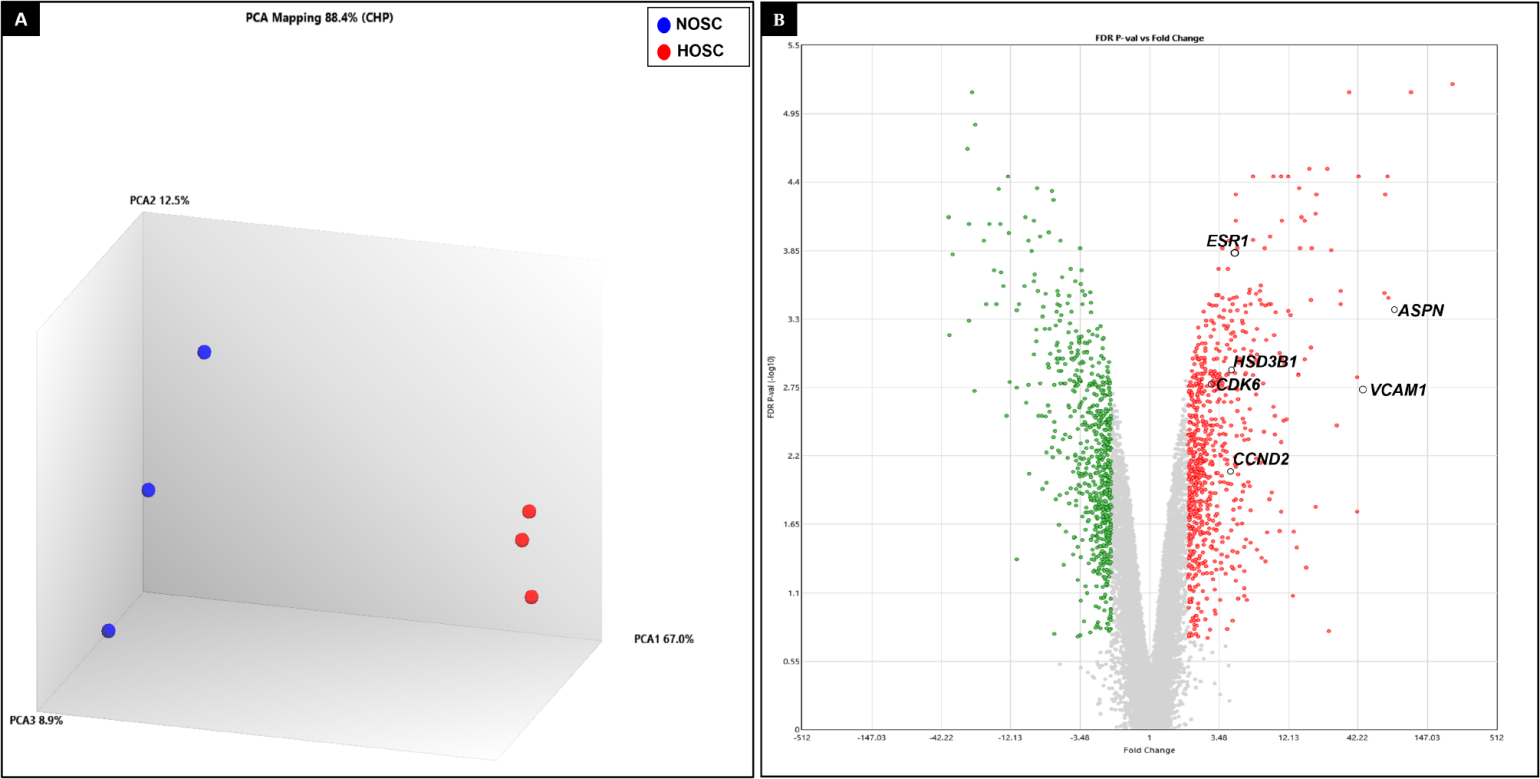
Principal component analysis and volcano plot. Unsupervised principal component analysis visualized differences in the transcriptomes of normal and hyperplastic ovarian stromal cells **(A)** Blue and red color dots denote individual samples from normal and hyperplastic ovary respectively. Each axis indicates the percentage of variation out of total mapped (88.4%) variation among samples. **(B)** Volcano plot of DE genes (*FDR p value* < 0.05, fold change ≤**-**2 or ≥ 2) between normal and hyperplastic ovarian stromal cells, red dots represents up-regulated genes and green dots represents down-regulated genes in HOSC. Important up-regulated genes such as *ASPN*,* VCAM1*,* HSD3B1*,* ESR1*,* CCND2* and *CDK6* expressed by HOSC are denoted on the volcano plot

**Fig. 7:**
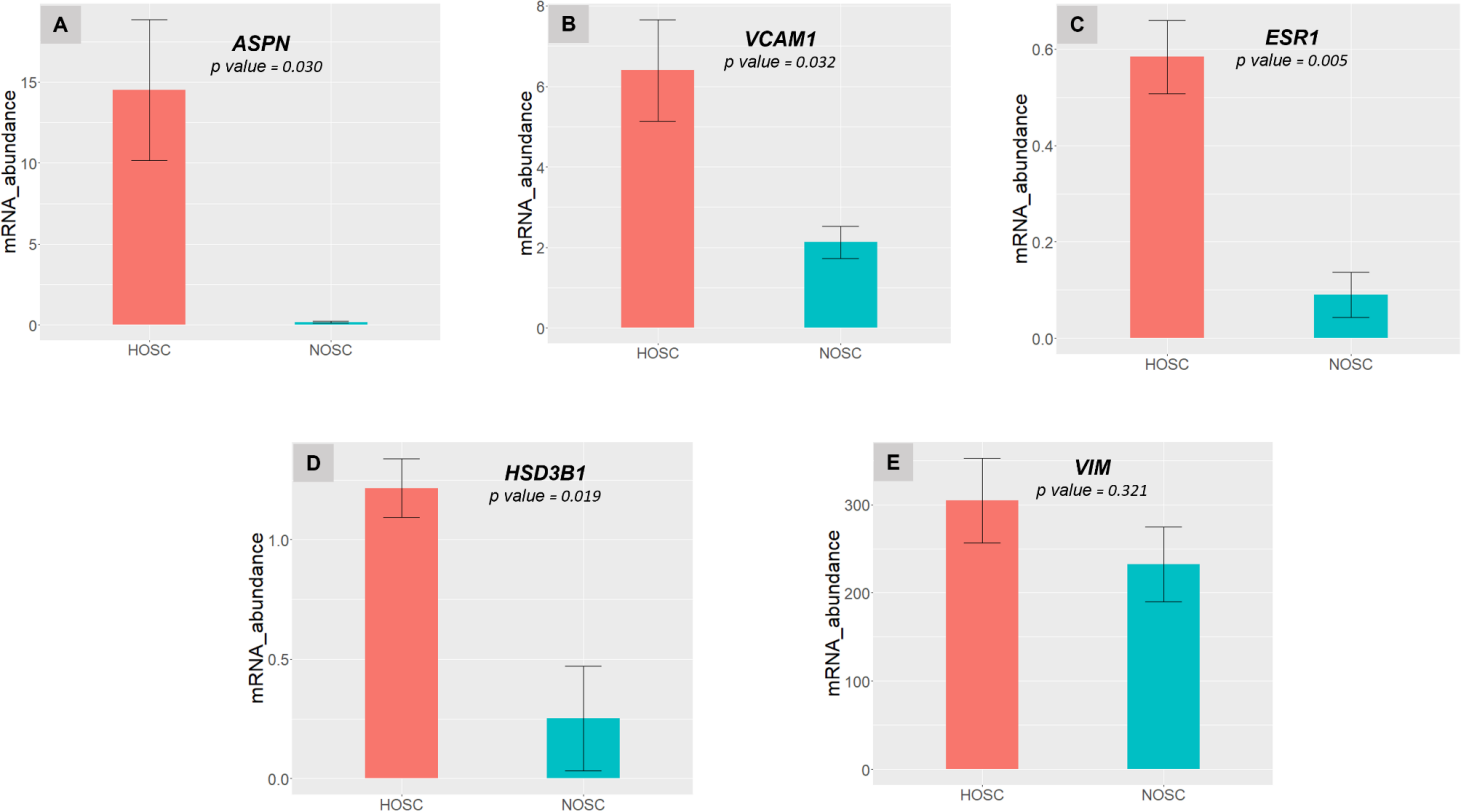
RT-qPCR validation of microarray data: **(A-E).** Bar plots represent gene expression of selected genes *ASPN*,* VCAM1*,* ESR1*, *HSD3B1* and *VIM* validated through RT-qPCR. Statistical significant changes were acknowledged with unpaired *t-test*, if *p* < 0.05. Data represented as means ± SEM, n=3

To investigate a possibly altered transcriptome of HOSC, we determined the mRNA expression profiles using microarray analysis across NOSC and HOSC. Evaluation of data from 3’ and 5’ hybridization controls (Supplementary image 4 and 5) and normalized signal box plots for all samples (Supplementary image 6) indicated that all array files were normal and passed the quality checkup. Unsupervised principal component analysis (PCA) was used to describe the clustering of samples based on the similarity of samples (Fig. 6A). Consequently, PCA of microarray sample data sets showed that NOSC and HOSC samples were distantly located from each other with PCA1 capturing 67% of variation and small variations of 12.5% and 8.9% were captured by PCA2 and PCA3 respectively. This indicated that a substantial difference existed in gene expression profiles of NOSC and HOSC samples. A total of 24,415 gene clusters were identified, out of which 1396 genes (733 were up- and 663 were down-regulated) were recognized as DE (FC ≤**-**2 or ≥ 2, *p* < 0.05, *FDR p* < 0.05) genes. The abundance of DE genes were visualized in a volcano plot as constructed with TAC 4.0 software (Fig. 6B). The volcano plot is plotted as FDR *p values* vs FC, where red and green dots represents differentially up- and down-regulated genes, respectively, while grey dots represent the unaltered genes. The top twenty up- and down-regulated genes in HOSC have been enlisted in Tables 1 and 2. Notably, asporin *(ASPN*; FC *=* 72.92, *q value =* 0.0003), insulin like growth factor-2 *(IGF-1*; FC *=* 71.84, *q value* = 3.58e-05), vascular cell adhesion molecule 1 *(VCAM1*; FC *=* 41.41, *q value* = 0.0015) and claudin 1 (*CLDN1*; FC *=* 30.68, *q value* = 0.0004) were strongly up-regulated. While, gap junction protein, alpha 5 (GJA5; FC*=-*23.34, *q value =* 0.0019), integrin, alpha 2 (ITGA2; FC*=-*16.4, *q value =* 0.0002) and integrin, alpha 6 (ITGA6; FC*=-*14.99, *q value =* 4.45E-05) were among markedly down-regulated genes in HOSC. Additionally, the mesenchymal cell marker gene *VIM* (*q value =* 0.144) was explicitly expressed by both NOSC and HOSC. This again confirmed the mesenchymal origin of both NOSC and HOSC. Also, in microarray data, collagen, type I, alpha 1 (*COL1A1)* gene an important fibroblast marker gene was equally expressed (*q value=0.99*) in both NOSC and HOSC, which clearly indicated that isolated stromal cells from both normal and tumor stromal tissue were stromal fibroblast cells. Further, five selected genes *ASPN*,* VCAM1*, estrogen receptor 1 (*ESR1)* and hydroxy-delta-5-steroid dehydrogenase, 3 beta- and steroid delta-isomerase 1 (*HSD3B1)* and *VIM* from microarray data were validated by RT-qPCR using RNA samples other than those analyzed by microarray analysis. The RT-qPCR results showed that mRNA expression of *ASPN* (*p value = 0.030*), *VCAM1* (*p value = 0.032*), *ESR1* (*p value = 0.005*) and *HSD3B1* (*p value = 0.019*) genes were significantly higher in HOSC as compare to NOSC (Fig. 7A and D). In addition, NOSC and HOSC equally expressed *VIM* (*p value = 0.321)*, the mesenchymal marker gene as also revealed by microarray data (Fig. 7E).

**Table 1:** List of top-20 up-regulated genes in HOSC

Affymetrix ID	Gene Symbol	Description	Tumor Avg (log2)	Normal Avg (log2)	Fold Change
12,705,367	EFEMP1	EGF containing fibulin-like extracellular matrix protein 1	11.41	3.56	230.63
12,780,150	ALPL	alkaline phosphatase, liver/bone/kidney	10.75	3.97	109.76
12,893,122	**ASPN**	asporin	9.91	3.72	72.92
12,829,389	IGF2	insulin-like growth factor 2	10.91	4.74	71.84
12,863,581	LOC100297676; LOC104970157	C-type lectin domain family 2 member D11; uncharacterized LOC104970157	8.84	2.73	69.04
12,859,375	A2M	alpha-2-macroglobulin	12.48	6.39	68.15
12,850,003	FGL2	fibrinogen-like 2	8.83	3.42	42.57
12,862,214	NDUFA4L2	NADH dehydrogenase (ubiquinone) 1 alpha subcomplex, 4-like 2	9.48	4.1	41.51
12,842,734	**VCAM1**	vascular cell adhesion molecule 1	8.25	2.88	41.41
12,766,526	LOC100850190; ULBP11; ULBP15; LOC100126815	NKG2D ligand 3-like; UL16-binding protein 11; UL16-binding protein 15; MHC class I-like family A1	6.74	1.57	36.21
12,889,029	OGN	osteoglycin	10.79	5.85	30.8
12,678,900	CLDN1	claudin 1	11.02	6.08	30.68
12,850,708	TSPAN13	tetraspanin 13	9	4.16	28.6
12,784,836	RANBP3L	RAN binding protein 3-like	7.07	2.42	24.99
12,780,587	TNFAIP6	tumor necrosis factor, alpha-induced protein 6	7.08	2.48	24.25
12,887,005	EBF1	early B-cell factor 1	8.59	4.28	19.86
12,718,310	SLPI	secretory leukocyte peptidase inhibitor	7.57	3.26	19.73
12,854,330	MEOX2	mesenchyme homeobox 2	7.63	3.34	19.64
12,902,661	LOC104976349	nik-related protein kinase-like	7.28	3.07	18.45
12,825,451	PDLIM3	PDZ and LIM domain 3	8.18	4	18.15

**Table 2:** List of top 20 down-regulated genes in HOSC

Affymetrix ID	Gene Symbol	Description	Tumor Avg (log2)	Normal Avg (log2)	Fold Change
12,870,136	GABRB1	gamma-aminobutyric acid (GABA) A receptor, beta 1	2.79	7.99	-36.89
12,680,761	GPX6	glutathione peroxidase 6 (olfactory)	4.99	10.19	-36.73
12,787,255	ADCY2	adenylate cyclase 2 (brain)	2.43	7.54	-34.55
12,908,119	CXHXorf57	chromosome X open reading frame, human CXorf57	2.54	7.26	-26.47
12,811,468	PRKCB	protein kinase C, beta	2.83	7.52	-25.75
12,696,603	GREM1	gremlin 1, DAN family BMP antagonist	3.85	8.54	-25.68
12,787,973	CDH6	cadherin 6, type 2, K-cadherin (fetal kidney)	3.18	7.79	-24.38
12,840,667	GJA5	gap junction protein, alpha 5, 40 kDa	4.02	8.57	-23.34
12,790,928	LRFN5	leucine rich repeat and fibronectin type III domain containing 5	3.31	7.83	-22.92
12,682,628	CYYR1	cysteine/tyrosine-rich 1	2.86	7.16	-19.67
12,721,596	HAS2	hyaluronan synthase 2	3.25	7.48	-18.8
12,822,597	KIAA1598	KIAA1598 ortholog	3.99	8.13	-17.73
12,786,958	ITGA2	integrin, alpha 2 (CD49B, alpha 2 subunit of VLA-2 receptor)	3.16	7.2	-16.4
12,775,831	TFPI	tissue factor pathway inhibitor (lipoprotein-associated coagulation inhibitor)	3.84	7.81	-15.61
12,783,018	ITGA6	integrin, alpha 6	5.45	9.35	-14.99
12,716,489	MYO3A	myosin IIIA	2.58	6.45	-14.61
12,723,184	SULF1	sulfatase 1	4.15	8.01	-14.47
12,732,196	CLMP	CXADR-like membrane protein	5.48	9.27	-13.79
12,757,828	CDH8	cadherin 8, type 2	3.89	7.61	-13.14
12,871,206	PARM1	prostate androgen-regulated mucin-like protein 1	4.5	8.17	-12.75

### GO terms and PPI network analysis

**Fig. 8:**
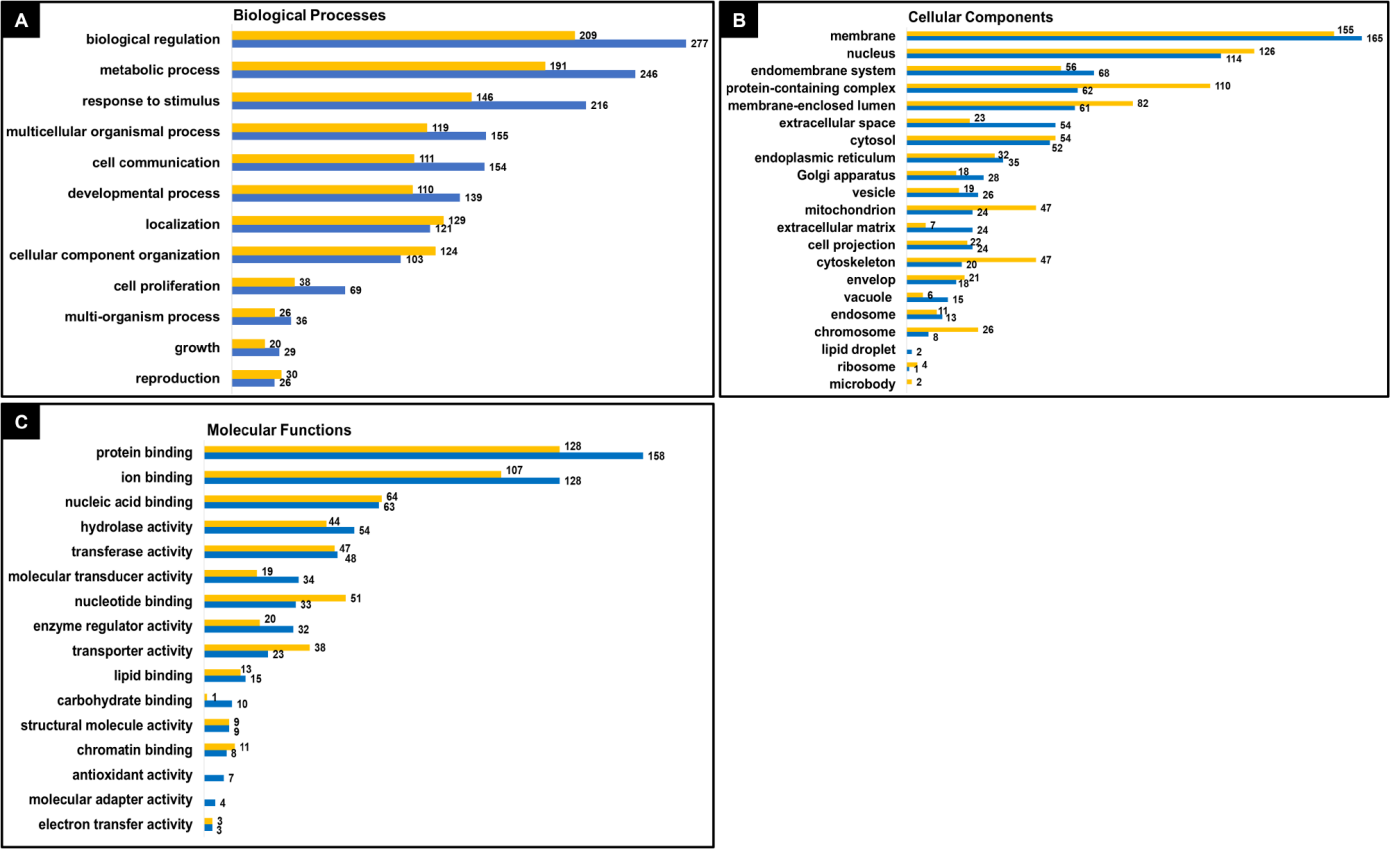
Gene ontology terms. DEGs in HOSC were subjected to GO slim annotations. The submitted genes were classified into **(A)** 12 biological processes, **(B)** 21 cellular components and **(C)** 16 molecular functions. Vertical axes indicate prioritized attributed functions. Blue bar and yellow bar represent up- and down-regulated genes respectively with numbers of genes allocated to each represented GO term

GO term analysis was carried out distinctly for up- and down-regulated genes using the WebGestalt tool. GO analysis was performed for the Bos *taurus* homologs of DE genes. The GO terms allocated functions to DE genes by priority to 12 biological processes, 21 cellular components and 16 molecular functions. Importantly, in biological processes, 246 up-regulated and 191 down-regulated genes represented “metabolic process” and around 68 up-regulated and 38 down-regulated genes were attributed to “cell proliferation” in HOSC (Fig. 8A). Among important cellular components, 110 down-regulated and 62 up-regulated genes were accredited to “protein containing complex” whereas 47 up-regulated and 24 down-regulated genes grouped to “mitochondria”. Notably, there were 24 up-regulated compare to 7 down-regulated genes identified to have function in “extracellular matrix” and around 47 down-regulated and 20 up-regulated genes in HOSC were related to “cytoskeleton” component of cells (Fig. 8B). Lastly, key molecular functions that were identified were “protein binding” and “ion binding” represented by 158 and 128 up-regulated and 128 and 107 down-regulated genes respectively. Similarly, “molecular transducer activity” was represented by 34 up-regulated and 19 down-regulated genes (Fig. 8C). Further, a zero order PPI network was identified among the DE genes to recognize the critical hub genes involved in HOSC. The resultant gene subnetwork 1 contained 104 nodes and 233 edges (Supplementary image 7). The hub genes were ranked based on their interacting degree and betweenness in PPI network (Supplementary sheet 2). Aurora kinase B (*AURKB)*, cyclin B2 *(CCNB2)* and kinesin family member 20 A *(KIF20A)* genes were ranked according to highest degree of 19, 18 and 18 respectively which indicates towards the direct connection these genes have with other genes in the network. Similarly, *ESR1*, epidermal growth factor receptor *(EGFR)* and cyclin *D1 (CCND1)* genes showed highest betweenness of 2403.58, 2265.08 and 1919.25 respectively, which clearly reflected the control these genes have over the entire sub-network (Table 3).

**Table 3:** List of top 25 hub genes

Gene Symbol	Degree	Betweenness	FC (HOSC/NOSC)
AURKB	19	143.39	-2.5
CCNB2	18	661.05	-2.63
KIF20A	18	226.84	-2.82
CCNB1	17	1591.7	-2.58
AURKA	17	104.83	-2.06
BUB1B	16	242.44	-2.33
CDC20	16	150.02	-2.77
KIF11	16	104.72	-2.02
PLK1	13	36.5	-2.7
KIF4A	12	75.89	-2.1
KIF2C	12	34.24	-2.59
NDC80	12	30.55	-2.26
ITGB3	11	1533.25	-5.53
CENPA	11	11.17	-2.48
PBK	10	28.22	-2.55
CDCA8	8	1.01	-2.06
ITGB8	7	27.25	2.69
ESR1	6	2403.58	4.88
EGFR	6	2265.08	-3.08
CDKN1A	6	1542.08	2.45
CDK6	6	1093.75	2.72
ACVR2A	6	203.5	2.07
NCAPG	6	0.35	-2.42
NCAPH	6	0.34	-2.19
CCND1	5	1919.25	-2.11

“Hub genes” are genes that have large interaction with many other genes; FC: expression fold change; HOSC: tumor ovarian stromal cells; NOSC: normal ovarian stromal cells

### Asporin, an extracellular matrix protein is strongly expressed in hyperplastic ovarian stromal tissue

**Fig. 9:**
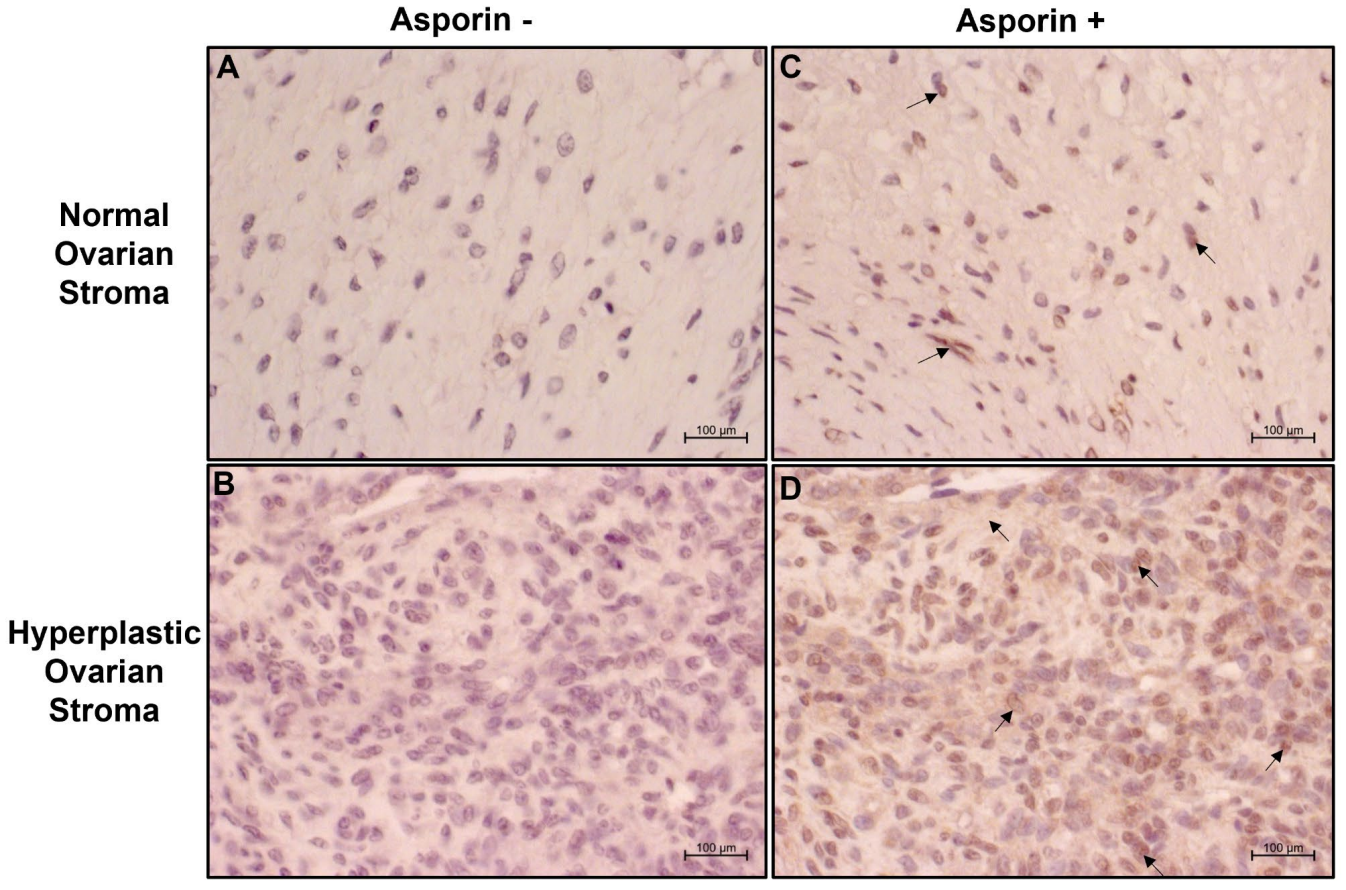
Representative immunohistochemically-labelled tissue sections of normal ovarian stroma and hyperplastic ovarian stroma stained for asporin protein. **(A)** normal ovarian stroma and **(B)** hyperplastic ovarian stroma represents negative controls (without anti-asporin antibody). Asporin (brown stains) was expressed relatively low in normal ovarian stroma **(C)** while increased expression observed in hyperplastic ovarian stroma **(D)**. Nuclei were counterstained with hematoxylin, images captured in LSM microscope at magnification 20x and scale bar: 100 μm

As revealed by both microarray and RT-qPCR data the mRNA expression of *ASPN* in HOSC was significantly higher compared to NOSC, so we reasoned whether asporin, an extracellular matrix protein [17] could be associated with altered proliferation of cells from hyperplastic stroma. The negative control for normal (Fig. 9A) and hyperplastic (Fig. 9B) ovarian stroma, without any primary antibody showed only hematoxylin stained nuclei. Upon staining with asporin primary antibody, normal stromal tissue showed a moderate positivity for asporin antibody (Fig. 9C). In contrast, an increased asporin expression (brown stains) was detected in the stroma of the hyperplastic ovary, with intense staining in stromal cell nuclei, cytoplasm and extracellular matrix (Fig. 9D). Together, these results suggest that asporin could be a plausible marker of an imbalanced stromal cell proliferation leading to a hyperplastic ovarian stromal tissue.

### Ingenuity pathway analysis (IPA)

To understand in-depth the functional changes induced in HOSC, enriched canonical pathways and upstream regulators were identified using the Ingenuity Pathway Analysis software (IPA; Qiagen Bioinformatics software solutions) based on Bovine Gene 1.0ST Arrays set as reference. The significance of association between the microarray data set and canonical pathways were determined from a *p-value* of overlap calculated using a right-tailed fishers exact test. A total of 643 enriched canonical pathways (Supplementary sheet 3) were identified and significantly enriched pathways (-log (*p value* ≥ 1.3) are presented in supplementary image 8. The corresponding *z-score* of each canonical pathway indicated the predicted activation or inhibited state of the pathway. A *z-score* ≥ 2 was considered significant. The significantly enriched canonical pathways with -log (*p value > 1.3)* with significant *z-score* ≥ 2 have been enlisted in Table 4. Of importance to tumor induction “Sirtuin Signaling Pathway” and “Mitochondrial Dysfunction” were found to be significantly activated canonical pathways in HOSC. Whereas, “Oxidative phosphorylation”, “RAC Signaling” and “Paxillin Signaling” were significantly inhibited canonical pathways in HOSC. Interestingly, “Molecular Mechanism of Cancer” (-log (*p value*= 5.14) and “Ovarian Cancer Signalling” (-log (*p value*= 2.68; *z-score*= -0.24) were also recognized as significantly enriched pathways but without any significant activation prediction.

**Table 4:** List of significantly enriched (activated/inhibited) canonical pathways in HOSC

Canonical Pathways	-log(*p*-value)	z-score	Molecules
Sirtuin Signaling Pathway	5.45	2.021	ACADL, APEX1,ARG2,ATG101,ATG13,ATP5F1B, ATP5F1D, ATP5MC1,ATP5PB, ATP5PF, BMAL1,CPT1A, CYC1,DOT1L, DUSP6,EPAS1,FOXO3,FOXO4,GABARAPL1,GABARAPL2,GADD45A, GADD45B, GADD45G, GOT2,GTF3C2,H1-5,H3C3,HSF1,IDH2,KAT2A, LDHB, MAP1LC3B, MAP1LC3C, MAPK12,MAPK4,NDUFA10,NDUFA12,NDUFA13,NDUFA2,NDUFA4,NDUFA4L2,NDUFB2,NDUFB7,NDUFS1,NDUFS6,NDUFS8,NDUFV1,PFKFB3,POLR1D, PPARG, PPARGC1A, PRKAA2,SDHA, SIRT6,SOD3,TIMM10,TIMM13,TIMM17A, TIMM22,TIMM23,TIMM44,TIMM9,TOMM40,TOMM5,TP53,TRIM28,TSPO, TUBA1B, TUBA1C, TUBA4A
Mitochondrial Dysfunction	4.33	2.092	ACADL, ACO2,AIFM1,APH1A, ARG2,ATP5F1B, ATP5F1D, ATP5MC1,ATP5PB, ATP5PF, CACNA1A, CACNA1G, CACNA1H, CACNA2D2,CACNB3,CALM1 (includes others), CAMK2A, CAPN6,CAPN7,CLIC2,COX10,COX5A, COX5B, COX7A1,COX7A2L, CYB5R3,CYC1,CYCS, DNM1L, FUS, GPX3,GPX6,GSTM1,HSD17B10,HSF1,IDH2,ITPR1,MAOB, MAP3K5,MAPK12,MCU, MGST1,NDUFA10,NDUFA12,NDUFA13,NDUFA2,NDUFA4,NDUFA4L2,NDUFB2,NDUFB7,NDUFS1,NDUFS6,NDUFS8,NDUFV1,PARK7,PDHX, PIK3CG, PIK3R3,PINK1,PPARG, PPARGC1A, PRKAA2,PRKAG1,PRKAR2A, PRKAR2B, PRKN, PTGES, RAPGEF3,SDHA, TARDBP, TOMM40,TP53,UQCR10,UQCRC1,UQCRQ
Oxidative Phosphorylation	3.03	-4.811	ATP5F1B, ATP5F1D, ATP5MC1,ATP5PB, ATP5PF, COX10,COX5A, COX5B, COX7A1,COX7A2L, CYC1,CYCS, NDUFA10,NDUFA12,NDUFA13,NDUFA2,NDUFA4,NDUFB2,NDUFB7,NDUFS1,NDUFS6,NDUFS8,NDUFV1,SDHA, UQCR10,UQCRC1,UQCRQ
Myelination Signaling Pathway	2.9	-2.047	ACVR2A, ACVRL1,ADGRG6,AKT3,APH1A, BDNF, BMP2,BMP4,BMP6,BMPR1B, CALM1 (includes others), CNP, DLC1,EGFR, EGR2,FASN, FGF2,FGFR1,FOS, FZD1,FZD4,FZD9,GLIS2,HCK, HDAC1,HDAC6,HDAC7,HDAC8,HDAC9,ID2,IGF1R, IGF2R, ITGA2,ITGA3,ITGA6,ITGB3,ITGB5,LAMA1,LAMA4,LAMB2,LMNB1,LYN, MLST8,MTHFD2,NRAS, NRG1,PDGFA, PDGFRA, PIK3CG, PIK3R3,PRKAG1,PRKAR2A, PRKAR2B, PTK2B, RAC1,RALB, RAP2B, RAPGEF3,RRAS, SMAD2,SMAD3,SMAD7,SMARCA4,TCF4,TSC2,WNT2,WNT4,WNT5A, WNT5B
Regulation of Actin-based Motility by Rho	2.78	-2.524	ACTG2,ACTR3,ARHGDIA, ARPC4,ARPC5L, BAIAP2,CFL1,DIRAS3,FNBP1,GSN, ITGA1,ITGA11,ITGA2,ITGA3,ITGA6,ITGA9,ITGAV, ITGB3,ITGB5,MYL6B, MYLK, PAK1,PAK3,PFN1,RAC1,RAC3,RND1,WASL, WIPF1
Mitotic Roles of Polo-Like Kinase	2.05	-2.714	ANAPC4,CCNB1,CCNB2,CDC20,CDC23,CDC25B, CDC25C, CDC27,CHEK2,ESPL1,KIF11,PLK1,PPP2R1B, PPP2R3A, PRC1,PTPA, SMC1A
tRNA Charging	1.65	-3.317	AARS1,FARSA, HARS1,LARS2,MARS1,NARS1,SARS1,SARS2,TARS1,VARS1,YARS1
RAC Signaling	1.53	-2.558	ACTR3,ARPC4,ARPC5L, BAIAP2,CD44,CFL1,ITGA1,ITGA11,ITGA2,ITGA3,ITGA6,ITGA9,ITGAV, ITGB3,ITGB5,NCKAP1,NRAS, PAK1,PAK3,PIK3CG, PIK3R3,PLD1,PTK2B, RAC1,RAC3,RALB, RAP2B, RRAS, SH3RF1,TIAM1
Paxillin Signaling	1.48	-2.183	ACTG2,ARHGEF7,CSK, ITGA1,ITGA11,ITGA2,ITGA3,ITGA6,ITGA9,ITGAV, ITGB3,ITGB5,MAPK12,NRAS, PAK1,PAK3,PIK3CG, PIK3R3,PTK2B, RAC1,RALB, RAP2B, RRAS, TLN1
Neutrophil Extracellular Trap Signaling Pathway	1.44	-4.341	AKT3,ARG2,ATP5F1B, ATP5F1D, ATP5MC1,ATP5PB, C1QB, CASP8,CERT1,COL11A1,COL15A1,COL18A1,COL3A1,COL4A1,COL4A2,COL4A5,COL4A6,COL8A1,CYC1,HCK, ITGA2,ITGA3,ITPR1,KCNN3,LYN, MAP2K3,MAPK12,MLKL, NDUFA10,NDUFA12,NDUFA13,NDUFA2,NDUFA4,NDUFA4L2,NDUFB2,NDUFB7,NDUFS1,NDUFS6,NDUFS8,NDUFV1,NLRP3,ORAI1,PIK3CG, PIK3R3,PLA2G4A, PLB1,PLCB4,PLCL1,PNPLA8,PRKCB, PRKCG, RAC1,RAC3,SDHA, STIM1,TIMM10,TIMM13,TIMM17A, TIMM22,TIMM23,TIMM44,TIMM9,TOMM40,TOMM5,TSPO
Glioma Invasiveness Signaling	1.35	-2.138	CD44,DIRAS3,FNBP1,ITGAV, ITGB3,NRAS, PIK3CG, PIK3R3,PLAU, PLAUR, RAC1,RAC3,RALB, RAP2B, RND1,RRAS, TIMP3
Granzyme A Signaling	1.34	3.357	APEX1,H1-5,LMNB1,NDUFA10,NDUFA12,NDUFA13,NDUFA2,NDUFA4,NDUFA4L2,NDUFB2,NDUFB7,NDUFS1,NDUFS6,NDUFS8,NDUFV1
Induction of Apoptosis by HIV1	1.34	-2.138	APAF1,BAK1,CASP8,CYC1,CYCS, DAXX, MAP3K5,MAPK12,NFKBIA, NFKBID, SLC25A13,SLC25A3,TNFRSF1B, TP53,TRAF1

The significantly enriched canonical pathways with -log (*p value > 1.3).* The corresponding *z-score* to each canonical pathway indicates the predicted activation or inhibited state of the pathway. Absolute *z-score* values ≥ 2 are considered significant, with an pathway considered as activated if the *z-score* is greater than or equal to 2 and inhibited if the *z-score* is less than or equal to − 2

**Fig. 10:**
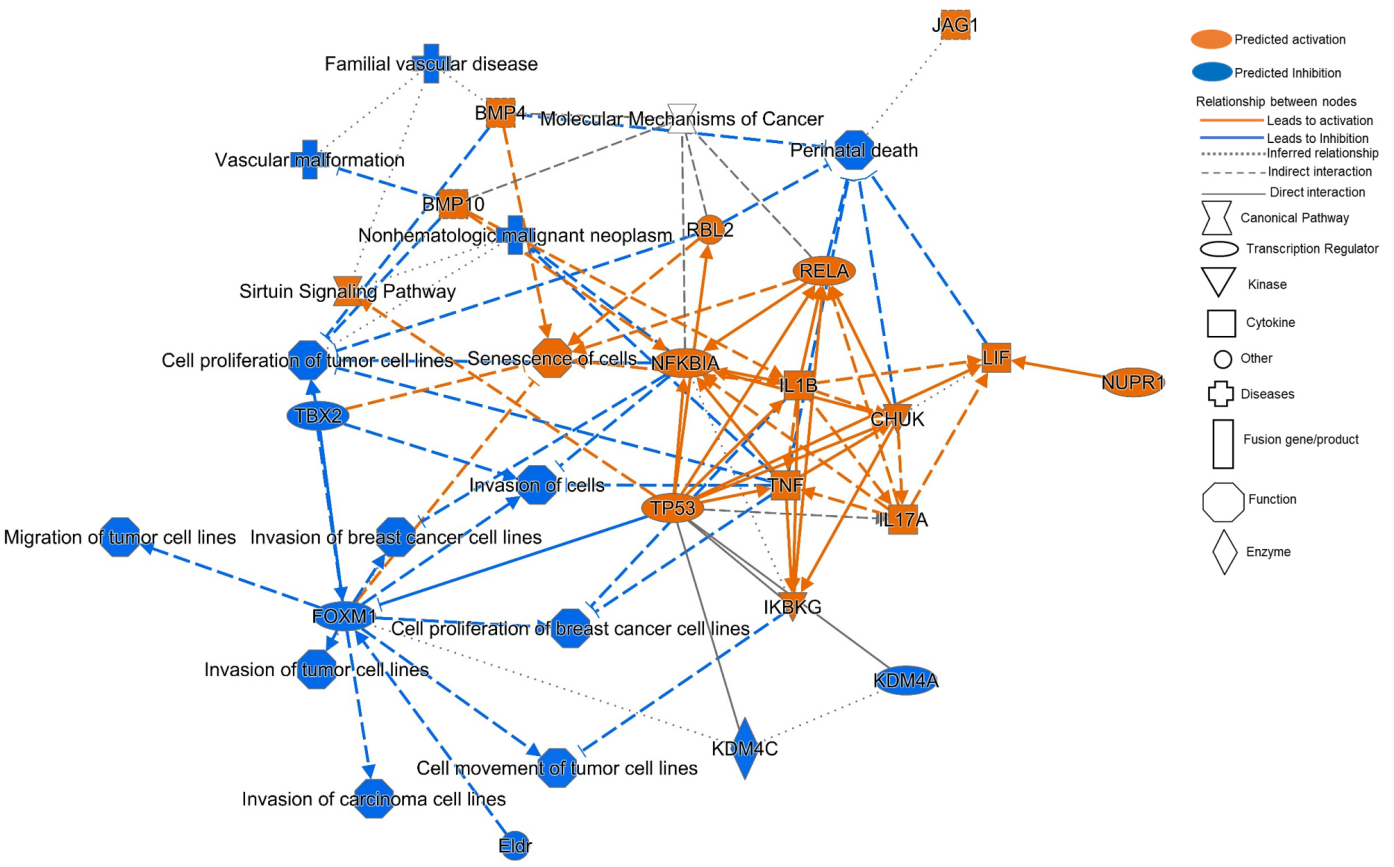
Graphical summary generated in IPA core analysis representing network of major canonical pathways, transcription factors and possible diseases and functions in HOSC

Further, upstream regulators with significant activation or inhibition with expression FC ≤ **-**2 or ≥ 2 are listed in Table 5. Importantly, NF-kappa-B inhibitor alpha *(NFKBIA)* was identified as significantly activated transcription regulators, while, peroxisome proliferator-activated receptor gamma coactivator 1-alpha (*PPARGC1)* was a significantly inhibited transcriptional regulator. An important receptor gene such as *ESR1* also emerged as highly inhibited upstream regulator. Surprisingly, tumor protein 53 (*TP53*) (FC=-1.47, *z-score*=5.72) was identified as highly activated upstream regulator by IPA (Supplementary sheet 4). IPA revealed several functions related to tumor phenotype that were highly decreased in HOSC such as “Cell proliferation of tumor cell lines”, “Cell movement of tumor cell lines”, “Migration of tumor cell lines”, “Invasion of cells” and “cell survival” (Supplementary sheet 5). Lastly, graphical summary generated by IPA represented major biological associations between key functional pathways such as “Molecular Mechanism of Cancer”, “Sirtuin Signaling Pathway” and upstream regulators. Activated upstream regulator NFKBIA was identified as direct target to transcription factors such as p65 (RELA) and TP53 and to growth factors such as tumour necrosis factor α (TNF) and Interleukin-1 beta (IL1B). Forkhead Box M1 (FOXM1), a major transcription factor was found to be directly inhibited by TP53, which could further passively lead to inhibition of aggressive tumor cell phenotypes such as cell migration, cell proliferation and cell invasion. This was certainly revealed by inhibition of several functions such as “Migration of tumor cell lines”, “Invasion of tumor cell lines” and “Cell proliferation of breast cancer cell lines”. Functional pathway of “Molecular Mechanism of Cancer” was indirectly influenced by activated NFKBIA, bone morphogenetic protein 10 (BMP10), RB transcriptional corepressor like 2 (RBL2) and RELA. Similarly, activated TP53 was indirectly associated to the “Sirutin signaling pathway” (Fig. 10).

**Table 5:** List of upstream regulators (activate/inhibited) in HOSC

Upstream Regulator	Expression Fold Change	Molecule Type	Prediction	z-score	*p*-value of overlap
ESR1	5.640	ligand-dependent nuclear receptor	Inhibited	-2.190	2.39E-08
NFKBIA	4.090	transcription regulator	Activated	3.063	1.14E-06
JAG1	3.520	growth factor	Activated	2.043	6.29E-07
SEMA7A	-3.280	transmembrane receptor	Activated	2.550	5.89E-03
mir-145	3.130	microRNA	Activated	2.356	4.75E-03
NRG1	-3.120	growth factor	Activated	2.146	4.17E-05
EGFR	-3.080	kinase	Inhibited	-2.167	1.50E-06
PRKAA2	2.680	kinase	Inhibited	-2.013	5.00E-03
BMP4	2.630	growth factor	Activated	2.148	3.09E-02
LAMA4	2.610	enzyme	Inhibited	-2.093	1.46E-04
PPARGC1A	2.600	transcription regulator	Inhibited	-4.697	8.32E-05
EGR1	2.570	transcription regulator	Activated	2.611	5.04E-03
MITF	2.530	transcription regulator	Inhibited	-2.084	5.94E-04
BHLHE40	2.390	transcription regulator	Activated	2.149	2.99E-03
ACTL6A	-2.230	other	Inhibited	-2.333	5.89E-03
H2AZ1	-2.110	other	Inhibited	-3.162	2.72E-02
KAT2A	-2.060	enzyme	Inhibited	-2.255	1.51E-02
HOXD8	2.030	transcription regulator	Activated	2.200	1.21E-02

The corresponding *z-score* to each upstream regulator indicates the predicted activation or inhibited state of the regulator

Absolute *z-score* values ≥ 2 are considered significant, with an upstream regulator being considered as activated if the *z-score* is greater than or equal to 2 and inhibited if the *z-score* is less than or equal to − 2

## Discussion

In the present study, we questioned if the hyperplastic ovary is related to alterations that could lead to risk of tumor progression. Surprisingly, the study revealed many cancer-associated genes that were found to be highly expressed in hyperplastic stromal cells as further discussed. In human benign tumors, hyperplastic cells proliferate significantly without turning malignant [18, 19]. Ovarian tumors in bovines are rare and earlier case studies have reported GCT in cattle with cystic follicles in the dissected section of an enlarged left-ovary [13]. However, in our study the cut section of the hyperplastic left-ovary revealed a uniformly distributed loosely held yellowish stromal tissue without any internal cystic follicles. The isolated cells from hyperplastic stromal tissue displayed spindle shaped fibroblast-like morphology *in vitro.* The HE stained stromal tissue showed increased number of densely packed stromal cells as compared to the HE stained stroma of a normal ovary. This finding supported our preliminary impression of increased proliferation of stromal cells that could have resulted in hyperplasia in the left-ovary. Additionally, significantly decreased expression of *ITGA2* (*q value*= 0.0002) and *ITGA6 (q value*= 4.53e-05*)* in HOSC could probably be the reason for loosely held connective tissue as observed in the hyperplastic ovary. Since, integrin’s are cell adhesion molecules that mediate cell-cell and cell-ECM interaction thus maintain a firm grip in matrix [20]. GCT in bovine may appear either as solid white tumor, cystic/solid tumor [21] or without severe lesions maintaining steroid receptor expression [22]. Contrary to this, our case study completely ruled out the condition of a solid or cystic GCT by the apparent appearance of loosely held connective tissue in the cut section of the hyperplastic left-ovary. Further, negligible amounts of E2 concentration were observed in spent culture media of HOSC and following microarray data showed very low mRNA expression of the granulosa cell key genes, E2 synthesizing enzyme cytochrome P450 Family 19 subfamily A member 1 (*CYP19A1)* and follicle-stimulating hormone receptor *(FSHR*). These results were indicative of non-estradiol synthesizing function of cultured HOSC. Further, cultured HOSC ceased to proliferate after eight passages. This behavior affirmed that cultured HOSC had a finite life span unlike malignant tumor cells that undergo uncontrolled proliferation in vitro [23]. The non-malignant phenotype of HOSC was also confirmed by a cell migration assay. It is well known that migrating cells such as cancer cells while migrating display their microtubule network as asymmetrical arrays [16]. In our study, the cultured HOSC while migrating across the 500 μm cell gap retained their normal symmetrical pattern. These in vitro cell migratory observations ruled out the notion of cancerous or metastatic nature of HOSC. Additionally, microarray data showed that the cell adhesion gene claudin 1 (*CLDN1*) (FC= 30.68, *q value*= 0.0004) was significantly up-regulated in HOSC, which could possibly put HOSC in risk of gaining migratory or invasive phenotype. As earlier reported, cell invasion and migration has been observed in normal liver and non-invasive human hepatocellular carcinoma due to overexpression of *CLDN1* [24]. Similarly, TNFAIP6 was highly up-regulated in HOSC. Interestingly, recent [25] reports have shown that over-expression of *TNFAIP6* in colorectal cancer cells results in tumor migration and invasion both in vitro and in vivo. NOSC and HOSC explicitly expressed VIM as validated by RT-qPCR and cell immunofluorescence analysis. These results confirmed the mesenchymal origin of both HOSC and NOSC and also verified NOSC as a reliable control to HOSC. Cultured normal bovine cortical/medullary stromal cells and human postmenopausal ovarian stromal cells are known to strongly express vimentin [26–28]. Recently, CAFs have gathered much attention as a potential cancerogenic source in many human stromal cancers. Quiescent CAFs [29] are known to display a fibroblast-like morphology, remarkably express *VIM*, and have a limited proliferation rate [30–32]. Further, RT-qPCR and microarray analysis confirmed a significantly higher expression of *ASPN* gene in HOSC than NOSC. ASPN is an extracellularly secreted protein that is reported to be predominantly expressed in stromal CAFs in malignant pathogenesis of human pancreatic [33], colorectal [34] and gastric cancers [35]. In mice, asporin protein was found to be expressed exclusively in the outer layer of theca cells of secondary follicles [36], however in our study, immunohistochemistry results revealed that asporin protein was not just limited to the outer layer of theca cells but also to the entire ovarian hyperplastic stroma. The presented results and earlier findings indicate that a high expression of *ASPN* in ovarian stromal cells could be an indicative marker gene of tumor progression in ovarian stroma. Apart from *ASPN*, RT-qPCR results confirmed higher *VCAM1*, *HSD3B1* and *ESR1* gene expression in HOSC. Earlier, *VCAM1* has been found to be strongly expressed in stromal cells of ovarian serous cystadenoma tissue [37]. Compared to theca cells, lower *HSD3B1* gene expression in human post-menopausal cultured stromal cells has been observed [28]. In the cultured HOSC, *HSD3B1* gene expression was comparatively higher to NOSC, but the corresponding P4 concentrations in both HOSC and NOSC were nearly equal. This suggest that plausibly hyperplastic stroma might have carried increased potential of expressing *HSD3B1* gene which could have led to increased androgen biosynthesis *via* cytochrome P450 family 17 subfamily A member 1 (*CYP17A1*) activation. This is indeed reflected by higher mRNA expression of *CYP17A1* (FC= 4.37, *q value*= 0.0489) in HOSC as revealed in microarray data. Additionally, it is known that ovarian stromal cells do express *CYP17A1* [38] and that higher androgen levels can induce exaggerated stromal hyperplasia [39]. To our knowledge, there are no sufficient reports on the *HSD3B1* gene involved in ovarian tumor progression. However, its role in primary tumors of 258 breast cancer patients showed that around 44% of patients were HSD3B1 positive and knockdown of HSD3B1 inhibited both cell proliferation and cell migration irrespective of estrogen receptor action [40]. In breast cancer cells, *ESR1* gene modulates S and G_2_/M phases of the cell cycle but in a ligand-dependent fashion [41]. However, in our study though *ESR1* was highly expressed in HOSC, negligible amounts of E2 were detected in spent media of cultured HOSC. Interestingly, the identification of ESR1 as a inhibited transcription factor by IPA and a major hub gene in network analysis, indicates an important role of ESR1 modulation in hyperplastic ovarian stroma. *ESR1* is a marker gene of E2 synthesizing cells such as granulosa cells and inhibition of ESR1 an upstream regulator as revealed by IPA could be the possible reason for negligible amounts of E2 secreted by HOSC. Contrary to this, a higher mRNA expression of *ESR1* in HOSC could be attributed to a ligand-independent action of ESR1 as previous studies in human endometrial cancer cells, have reported higher estrogen-independent activity of ESR1 in D538G mutants compare to their wild-type counterparts [42]. Additionally, cyclins such as CCND2 are critical in promoting G1/S cell cycle progression and inducing tumorgenesis in mice glioblastoma stem cells [43]. The microarray array mRNA expression signals for *CCND2* (FC=3.85; *q value* =0.0067) and *CDK6* (FC=2.72; *q value* =0.0013) were significantly higher in HOSC. This is in line with higher proportions of HOSC observed in the S-phase of cell cycle during flow cytometry analysis as both CCND2 and CDK6 are important regulators of G1/S phase of cell cycle [44]. Also, a higher expression of *CCND2* is reported to induce proliferative, migratory and invasive behaviour in human ovarian cancer cells [45]. Activation of *ESR1* increases *CCND2* expression and induces proliferation of human testicular embryonal carcinoma NT2/D1 cells [46]. Together, this ligand independent action of *ESR1* could be anticipated in HOSC proliferation *via* up-regulation of *CCND2* which could have led to an over-amplified population of stromal cells in hyperplastic ovarian stroma *in vivo.* An additional key player could be *NFKBIA*, which is known to induce cell proliferation and inhibit apoptosis [47, 48] and was highly activated in HOSC as revealed by microarray data and IPA. However, effector molecules involved in an E2-independent action of Erα (*ESR1*) activation involved in cell proliferation in hyperplastic ovary still need further investigation. Sirtuins (SIRTs 1–7) are known to be differentially expressed in several human cancers [49]. Lower expression of *SIRT6* is known to suppress the proliferation of human pancreatic ductal adenocarcinoma (PDAC) cells *via* histone deacetylation [50]. Interestingly, in our microarray data *SIRT6* expression was significantly (FC=-1.51, *qvalue=*0.032) lower in HOSC indicating that SIRT6 might have contributed in restricting the aggressive proliferation of HOSC in the hyperplastic stroma. However, the role of SIRT6 in tumor progression is still unclear as SIRT6 can act either as a tumor promoter or tumor suppressor depending on the status of the tumor tissue. It is well known that, glucose metabolism is reprogrammed during tumorigenesis for a rapid growth and survival, so tumor cells switch to glycolysis instead of oxidative phosphorylation (OXPHOS) [51]. 6-phosphofructo-2-kinase/fructose-2, 6-biphosphatase 3 (PFKFB3) is one of the critical enzyme of the glycolytic pathway and an increased mRNA expression of *PFKFB3* is well documented in aggressive neoplasm of ovary, colon and breast [52] and in gastric cancer tissue [53]. Similarly, our microarray data, revealed higher mRNA level of *PFKFB3* (FC*=*4.71, *qvalue=0.0018*) in HOSC as compared to NOSC. Also, the majority of genes involved in OXPHOS, including cytochrome C1 (*CYC1)* (FC=-2, *qvalue*=0.0057) and cytochrome C5 (*CYC5)* (FC=-2.62, *qvalue*=0.0035) were downregulated in HOSC. Apart from glycolysis, PFKFB3 is also known to stimulate cell proliferation in cancer cells as revealed by the knockdown of *PFKFB3* which suppressed both glycolysis and cell-cycle G1/S progression in renal cell carcinoma cell lines [54]. *PPARGC1* is a key regulator of mitochondrial biogenesis [55] and its inhibition might contribute in mitochondrial failure. Together, these findings clearly indicate that inhibition of OXPHOS and active glycolysis might have been readily used for ATP synthesis by hyperplastic stromal tissue to maintain HOSC in a viable and proliferative state. Tumor cells with mitochondrial dysfunction have increased reactive oxygen species (ROS) levels compared to normal cells, and cell apoptosis is the outcome of the cells under ROS stress unless the stressed cells are rescued by upregulation of oxidative stress response genes such as superoxide dismutase (SOD) and glutathione peroxidase 1(GPx) [56]. The involvement of stress response genes in regulating apoptosis in HOSC were confirmed through higher expression levels of stress response genes such as *SOD3* (FC=4.66, *qvalue*=0.0141) and *GPX3* (FC=6.81, *qvalue*=0.0006) suggesting the role of stress response genes in rescuing HOSC from undergoing apoptosis in spite of disruptive mitochondrial OXPHOS.

## Conclusion

The molecular alterations contributing to ovarian hyperplasia are challenging to investigate as hyperplasia is often either ignored or goes unnoticed in field conditions. Moreover, the affected animals are subjected to slaughter due to poor reproductive performance. In this case study, we observed a Holstein cow with ovarian hyperplasia and collected its abnormally enlarged ovary for in vitro characterization for the first time. We identified that non-steroidogenic stromal cells are significant contributors to ovarian hyperplasia in the studied animal. Suggestively, altered expression of extracellular matrix proteins, cell adhesion and cell proliferation genes in stromal cells might induce a growth imbalance leading to the abnormal enlargement of ovarian stromal tissue. However, realizing the constraints of limited sample size in the presented case study, further investigation with a larger sample size is needed to strongly verify this relation which might contribute to a better understanding of the physiology of ovarian hyperplasia in bovine and related species.

## Electronic supplementary material

Below is the link to the electronic supplementary material.

Supplementary Material 1

Supplementary Material 2

Supplementary Material 3

Supplementary Material 4

Supplementary Material 5

Supplementary Material 6

Supplementary Material 7

Supplementary Material 8

Supplementary Material 9

Supplementary Material 10

Supplementary Material 11

Supplementary Material 12

Supplementary Material 13

## Data Availability

No datasets were generated or analysed during the current study.
