# Supplementary figures and images for: Hyperplastic ovarian stromal cells express genes associated to tumor progression: a case study
Source: BMC Vet Res. 2024 Sep 28;20:439. doi: 10.1186/s12917-024-04275-6 (PMC11438404; doi:10.1186/s12917-024-04275-6)

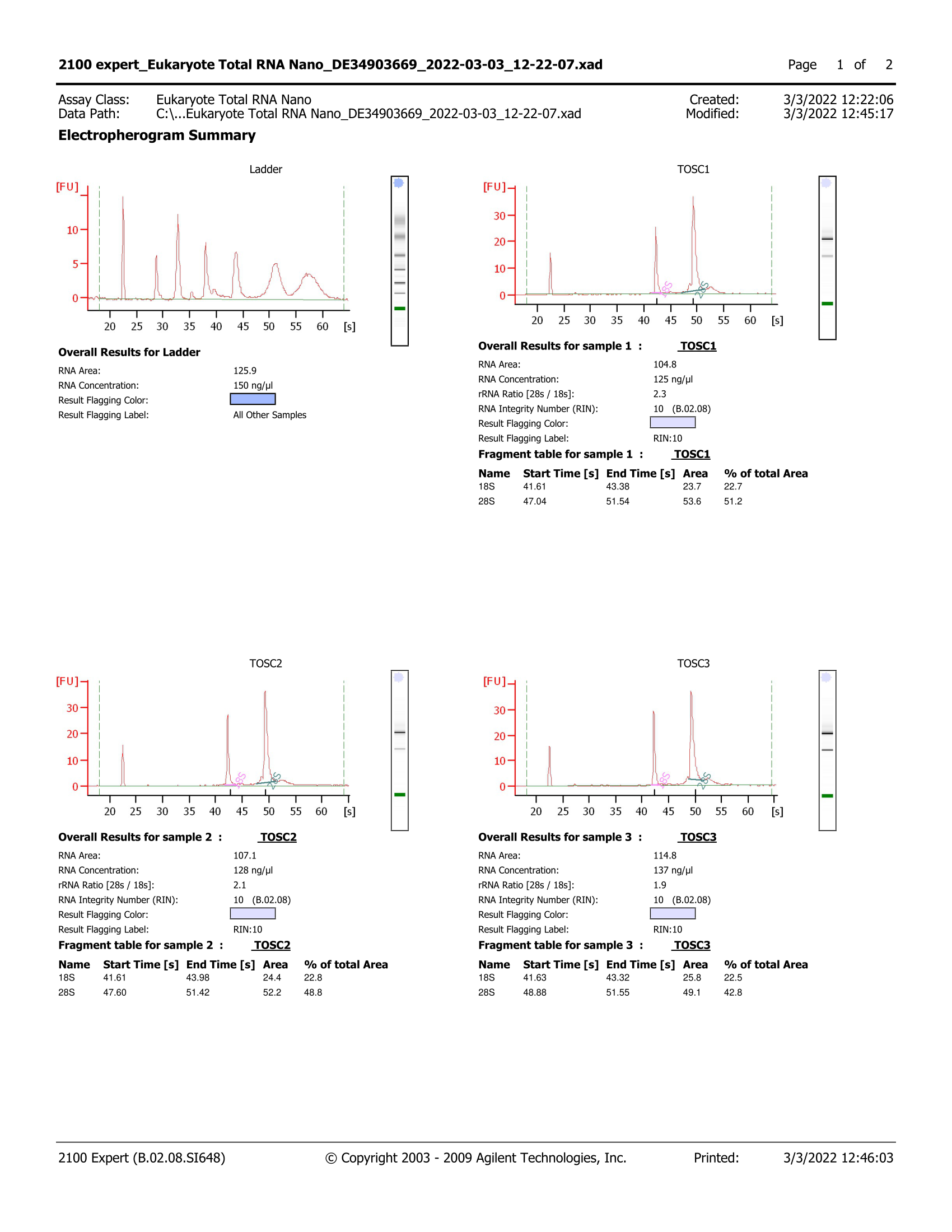

Supplement: Supplementary file 1 — Supplementary Material 1 [file 12917_2024_4275_MOESM1_ESM.tiff]

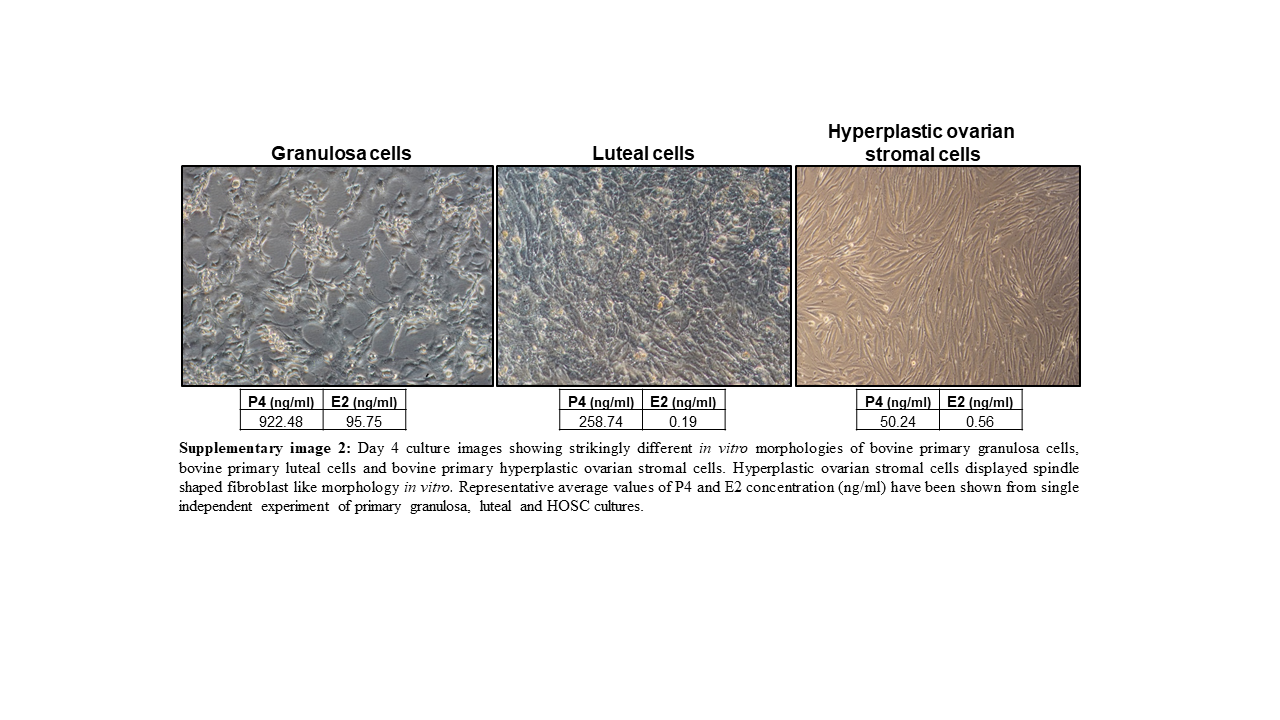

Supplement: Supplementary file 2 — Supplementary Material 2 [file 12917_2024_4275_MOESM2_ESM.tif]

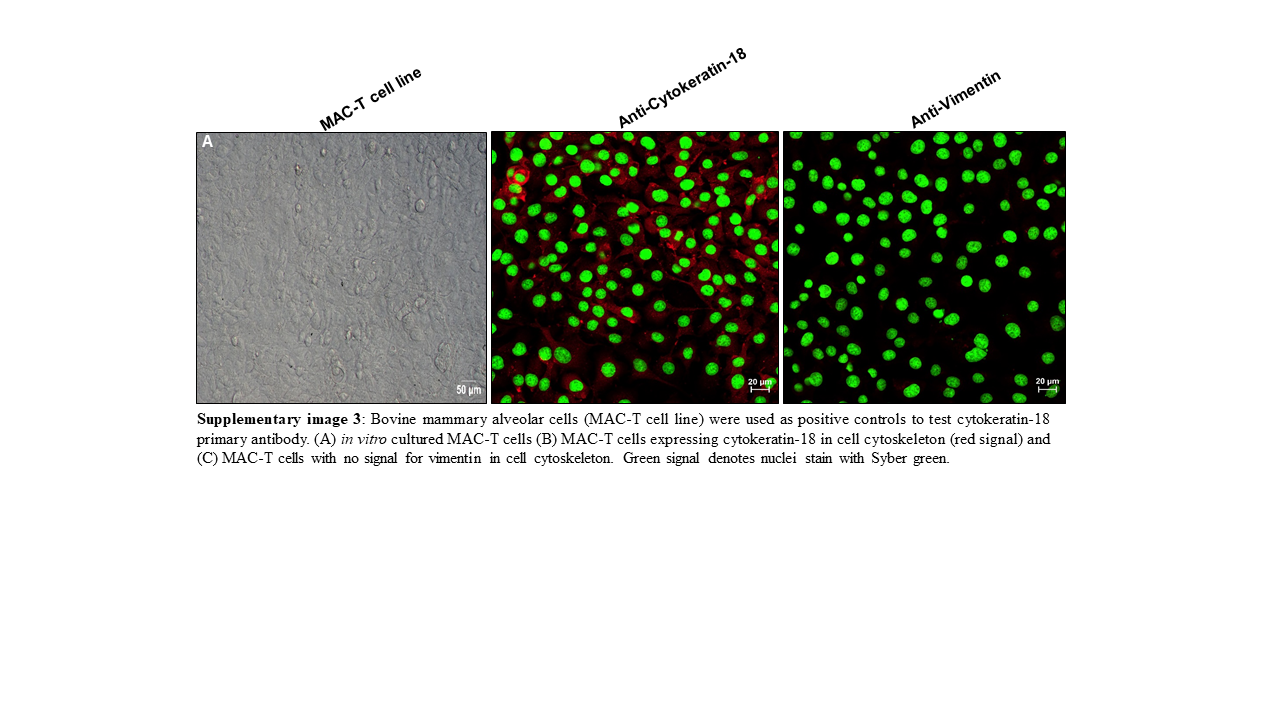

Supplement: Supplementary file 3 — Supplementary Material 3 [file 12917_2024_4275_MOESM3_ESM.tif]

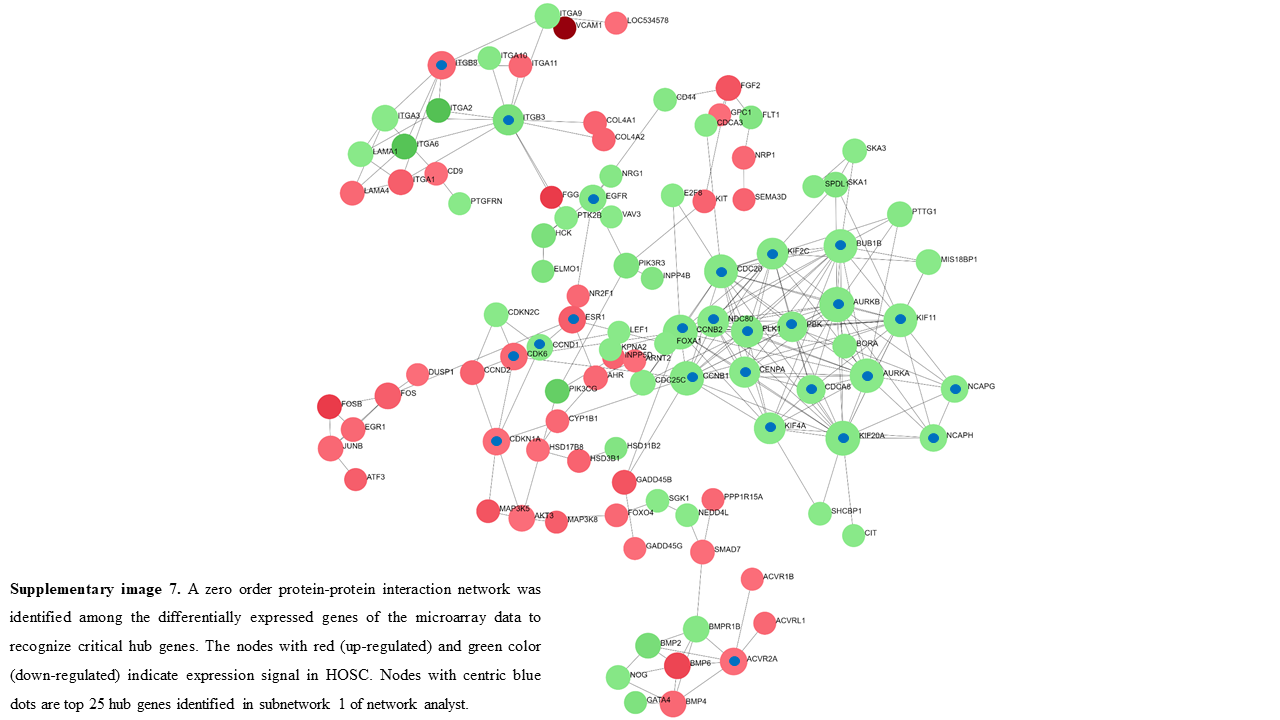

Supplement: Supplementary file 7 — Supplementary Material 7 [file 12917_2024_4275_MOESM7_ESM.tif]

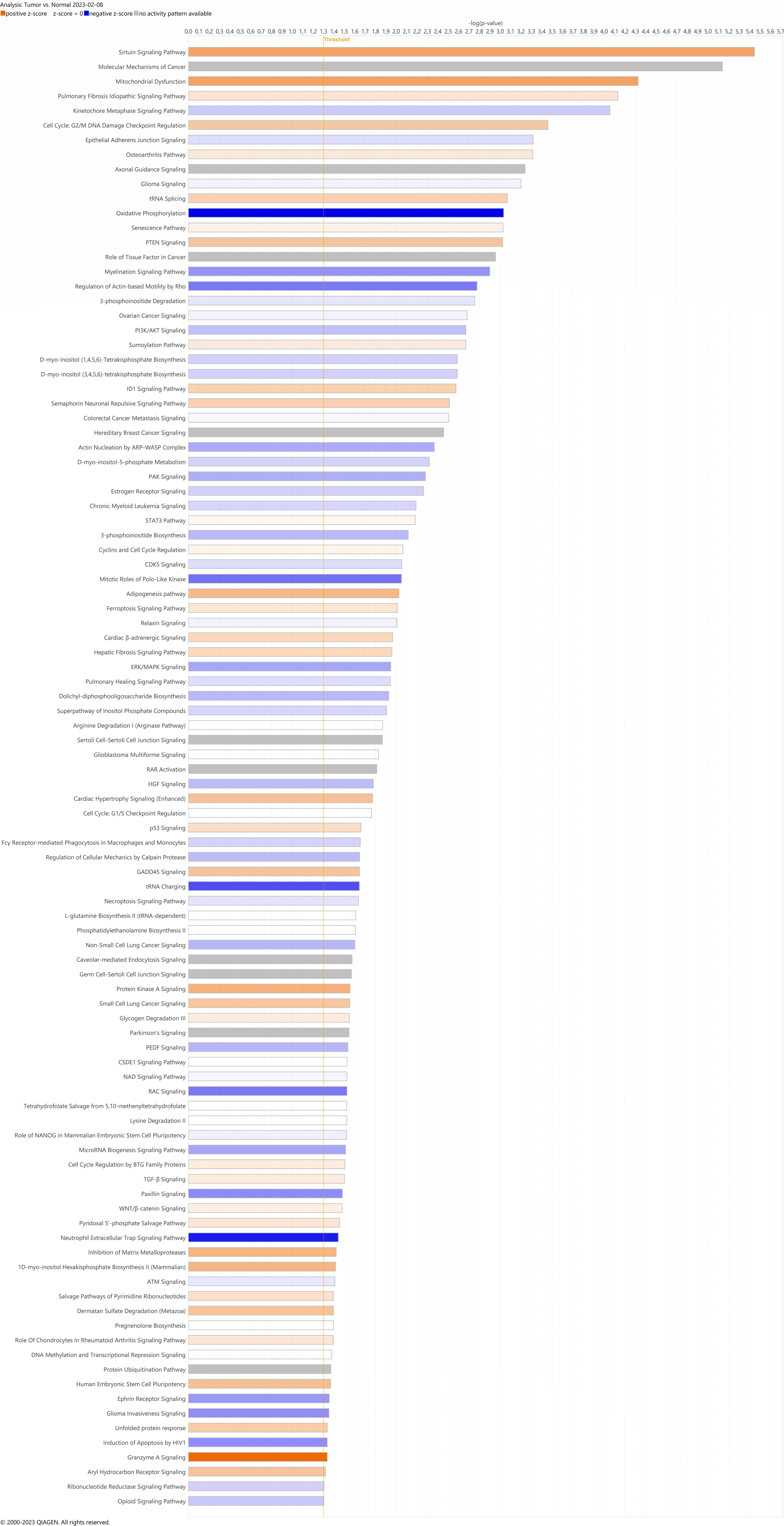

Supplement: Supplementary file 8 — Supplementary Material 8 [file 12917_2024_4275_MOESM8_ESM.tiff]
