# Supplementary material for: Hyperplastic ovarian stromal cells express genes associated to tumor progression: a case study
Source: BMC Vet Res. 2024 Sep 28;20:439. doi: 10.1186/s12917-024-04275-6 (PMC11438404; doi:10.1186/s12917-024-04275-6)
Supplement: Supplementary file 9 — Supplementary Material 9 [file 12917_2024_4275_MOESM9_ESM.docx]

**Supplementary Sheet 1: List of Primers:**

| **Primers** | **Sequence** | **Length (bp)** | **Accession Number** |
| --- | --- | --- | --- |
| **ASPN** | **Forward:** AGGTCTGTCCTCCGTCCCAAGCAA  **Reverse:** ACCCCTTCAAATGCCCCTGGTTCA | **387** | **NM_001034309.2** |
| **VCAM1** | **Forward** :TGAGGGGACCACATCCATGCTGA  **Reverse:** TGGGACGGTTGCCTTTCAACAACT | **257** | **NM_174484.1** |
| **VIM** | **Forward:** GGATGCGCTCAAAGGGACTA  **Reverse:** CCAGATTGGTTTCCCTCAGGT | **296** | **NM_173969.3** |
| **ESR1** | **Forward:** CTGCCAAGGAGACTCGCTAC  **Reverse:** CCTCCTCTTCGGTCTTTCCG | **253** | **NM_001001443.1** |
| **HSD3B1** | **Forward:** TGTTGGTGGAGGAGAAGGATCTG  **Reverse:** GCATTCCTGACGTCAATGACAGAG | **208** | **NM_174343** |
| **TBP** | **Forward:** GCCTTGTGCTTACCCACCAACAGTTC  **Reverse:** TGTCTTCCTGAAACCCTTCAGAATAGGG | **200** | **NM_001075742** |
